# Vehicular driving pattern analysis: concepts, challenges, and future directions

**DOI:** 10.3389/frai.2026.1931751

**Published:** 2026-10-28

**Authors:** Raparthi Yaswanth, M. Rajasekhara Babu

**Affiliations:** School of Computer Science and Engineering, Vellore Institute of Technology, Vellore, Tamil Nadu, India

**Keywords:** AR/VR simulation, biometric monitoring, driver behavior analysis, federated learning, intelligent transportation systems, real-time monitoring

## Abstract

**Introduction:**

Vehicular driving pattern analysis is important for improving road safety, driving efficiency, and intelligent transportation systems. The aim of this study is to analyze recent research on the major driver-behavior analysis tasks, which are driving pattern recognition, distraction detection, abnormal driving-behavior detection, driver profiling and identification, fuel-efficiency analysis, and driving-risk assessment.

**Methods:**

The literature selection process was structured and included identification, screening, and eligibility. One hundred seventeen studies were used in this study, and 12 studies were selected for a detailed examination for comparative analysis.

**Results:**

The studies analyzed have shown that driver-behavior analysis is becoming more popular with the use of machine learning, deep learning, sensor fusion, edge computing, and other new technologies. Existing approaches, however, are still scattered across behavioral tasks and encounter issues such as real-time processing, privacy preservation, scalability, model generalization, and integration of heterogeneous data.

**Discussion:**

This study highlights the need for integrated and task-oriented approaches to address these challenges and support multiple behavioral assessments. The findings provide research directions on the development of scalable privacy-aware real-time driver-behavior analysis systems for connected and intelligent transportation environments.

## 1 Introduction

The automotive sector has undergone significant technological advances, leading to smarter vehicles and sophisticated systems for monitoring and analyzing driver behavior. These advances have enabled sensor-based monitoring, machine learning, and real-time data analytics to assess and predict driver behavior, thereby supporting road safety, insurance modeling, and driving efficiency. Despite these advances, accurately analyzing and responding to driver behavior in real time remains challenging. Accurate identification of driving patterns, risk assessment, and personalized interventions are increasingly important (Bustos et al., 2025). Analysis of driver behavior can play a crucial role in enhancing safety results, reducing fuel use, and allowing the design of intelligent transportation systems. With the growing connection and autonomy of vehicles, real-time feedback of driver behavior will become central in making transportation safer and more efficient (Liu et al., 2025). The study is aimed at exploring the ways driver behavior may be examined with the help of advanced technologies to deliver quantifiable improvements to safety, efficiency, and sustainability.

### 1.1 Research motivation

The growing sophistication of contemporary transport systems and the continued focus on road safety and fuel efficiency, as well as on the development of autonomous vehicles, have intensified the necessity of accurate and real-time driver behavior analysis. Conventional solutions tend to concentrate on single elements like detecting distraction or profiling driving styles and do not have the scalability and flexibility needed to apply in practice. Additionally, with the increasing presence of connected and intelligent vehicles, there is an acute need for systems that would be able to consume different sensor data, preserve the privacy of the driver, and perform well in different road and traffic conditions. This inspires the creation of a single, smart system capable of combining various behavior-related activities with the help of such high-tech solutions as machine learning, federated learning, edge computing, and even immersive AR/VR-based models. The gap needs to be tackled to improve the safety of driving, provide more personalized models of insurance, allow predictive maintenance, and serve the greater purpose of sustainable and intelligent transportation systems as outlined in Table 1.

**Table 1:** Summary of related work.

Research	Contributions	Limitations
Cheng et al., 2025	Provides a comprehensive survey on driving safety risks in mixed traffic environments. Identifies critical risk factors associated with connected and non-connected vehicles. Discusses potential countermeasures to enhance road safety.	Lacks real-world implementation details of proposed safety measures. Does not extensively cover emerging AI-driven risk assessment models.
Ju and Li, 2024	Reviews EEG-based methods for detecting driver states and behaviors. Covers a wide range of applications, including drowsiness and cognitive load detection. Highlights challenges in real-time EEG-based driver monitoring.	Provides limited discussion on practical deployment challenges in real-world driving conditions. EEG signal variability across individuals is not fully addressed.
Ahmed et al., 2025	Explores AI-driven advancements in remote photoplethysmography (rPPG) for driver monitoring. Compares various AI models and their effectiveness in physiological monitoring. Identifies future research directions in rPPG-based driver monitoring.	Limited real-world testing and validation of AI-driven rPPG systems. Challenges related to environmental factors affecting rPPG accuracy are not fully addressed.
Gyllenhammar et al., 2025	Reviews methodologies for ensuring the safety of automated driving systems. Discusses regulatory and certification frameworks for autonomous vehicles. Identifies gaps in current safety validation techniques.	Primarily focuses on regulatory perspectives rather than technical implementations. Limited discussion on cybersecurity threats in automated driving.
Tan et al., 2024	Surveys various techniques for detecting driver distractions in autonomous driving. Compares available datasets for driver distraction recognition. Highlights challenges in real-time distraction detection.	Lacks an in-depth evaluation of multimodal fusion techniques for distraction recognition. Limited real-world testing of distraction detection models.
Liao et al., 2024	Discusses the importance of personalization in driving behavior modeling. Reviews existing datasets and their suitability for personalized models. Identifies gaps in current validation techniques for personalized driver models.	Limited discussion on the ethical implications of personalized driving models. Challenges in real-time implementation of personalized driving models are not fully explored.
Gupta et al., 2024	Investigates deep learning applications for driver behavior detection. Highlights the role of cyber-physical systems in intelligent transport. Compares different deep learning models for behavior classification.	Computational complexity of deep learning models is not fully addressed. Limited focus on interpretability and explainability of deep learning models.
Fu et al., 2024	Provides an extensive review of modern drowsiness detection methods. Compares different sensor modalities used in drowsiness detection. Identifies gaps in existing research and suggests future directions.	Limited discussion on the integration of multiple sensor modalities. Real-time deployment challenges are not comprehensively addressed.
Bouhsissin et al., 2023	Categorizes driver behavior classification approaches. Highlights the impact of driver behavior on road safety. Identifies gaps in the current classification methods.	Limited discussion on data imbalance issues in behavior classification datasets. Focuses on traditional classification methods rather than emerging deep learning techniques.
Wang et al., 2021	Reviews in-vehicle camera-based techniques for driver behavior analysis. Discusses challenges in using computer vision for behavior monitoring. Identifies potential improvements in vision-based driver monitoring systems.	Privacy concerns related to in-vehicle camera monitoring are not deeply explored. Challenges related to varying lighting conditions affecting camera performance are not fully addressed.
Our paper	This study explores the integration of artificial intelligence in modern transportation systems, focusing on vehicle data analysis and intelligent decision-making. It highlights the role of AI-driven methodologies such as predictive analytics, reinforcement learning, and anomaly detection in enhancing vehicle health monitoring, optimizing traffic flow, and improving accident prevention strategies. The study provides insights into how AI can be leveraged to create a more efficient and safer driving environment by enabling real-time data processing and adaptive learning mechanisms. Furthermore, it discusses the implementation of AI models in autonomous and semi-autonomous vehicles, emphasizing their potential to revolutionize connected ecosystems by facilitating vehicle-to-vehicle and vehicle-to-infrastructure communication. This study also addresses the significance of AI in enhancing situational awareness and proactive decision-making in dynamic driving conditions.	

### 1.2 Research objectives and contributions

The main aim of this study is to compare the existing methods of analyzing the behavior of drivers with particular attention paid to essential activities, including detection of distraction, abnormal driving patterns, evaluation of fuel efficiency, and risk assessment. The research will try to determine the shortcomings of the existing approaches, especially the lack of cohesive, scalable, and real-time systems that can manage various tasks involving behaviors (Masello et al., 2025). It aims to consider how well-developed technologies such as machine learning, deep learning, sensor fusion, and biometric monitoring can be used to improve behavioral prediction and safety outcomes. Furthermore, the study examines how emerging technologies like federated learning, edge computing, blockchain, and AR/VR can be used to address the current problems. Lastly, the study explains a future architecture which incorporates the above technologies in order to provide secure, adaptive, and privacy-sensitive driver behavior analysis systems.

This study makes a number of important contributions to the sphere of driver behavior analysis. It presents a task-focused overview of the current research, encompassing the major fields of the research like distraction detection, abnormal behavior recognition, fuel efficiency analysis, driver identification, and insurance risk prediction (Shim et al., 2025). The study provides a comparative assessment of the different machine learning and deep learning strategies focusing on effectiveness and limitations concerning different tasks. It also plots the datasets and performance metrics employed in the state-of-the-art studies, which provides a summary of the present evaluation environment. Notably, the study also found a significant research gap, which is the unavailability of an integrated, scalable, and real-time framework that would be able to work on various behavior analysis tasks at the same time. The study will aim to solve this by suggesting a future architecture that will include federated learning, privacy protection, edge computing, real-time responsiveness, and AR/VR-based simulations to model and train driver behavior.

This review differs from current reviews of driver behavior analysis literature, which tend to be more specific to a particular aspect of driver behavior, by offering a holistic and task-oriented review of the field of distraction detection, abnormal driver behavior, fuel efficiency, driver identification, and risk assessment. It further connects these tasks to emerging technologies such as federated learning, edge computing, sensor fusion, blockchain, and AR/VR and proposes the need for a scalable, real-time, privacy-conscious, and unified approach to comprehensive analysis of driver behavior.

#### 1.2.1 Comparison with recent reviews and distinct contribution

Review studies have recently focused on specific aspects of driver behavior analysis. Bouhsissin et al. (2023) studied driver behavior classification, and Wang et al. (2021) focused on driver behavior analysis based on cameras installed in the vehicles. Driver distraction recognition has been reviewed in (Tan et al., 2024), and personalization in driving behavior in (Liao et al., 2024), deep-learning driver behavior detection in (Gupta et al., 2024), and drowsiness detection in (Fu et al., 2024) have all been reviewed. The studies described in Sections 2.2–2.5 most often involve a particular behavioral task, sensing modality, or modeling approach. This review, on the other hand, offers a wider task-oriented analysis that includes driving pattern recognition, the detection of distractions, the detection of abnormal driving behavior, driver profiling and identification, fuel-efficiency analysis, and driving-risk assessment. The review also covers new technologies like federated learning, edge computing, sensor fusion, blockchain, and AR/VR, alongside their implications for real-time processing, privacy, scalability, and personalized feedback for drivers. This wider context helps form a comprehensive overview of the review, linking behavioral tasks, technologies to support the tasks, applications, issues, and future research directions.

This review focuses primarily on driver-behavior analysis, such as driving pattern recognition, detection of driver distraction, detection of abnormal driving behaviors, driver profiling and driver identification, fuel-efficiency analysis, and driving-risk assessment. Autonomous-driving perception, traffic management, vehicle maintenance, and cybersecurity are considered only if they directly support or interact with driver-behavior analysis. However, autonomous perception like object detection, traffic-sign recognition, and environment perception is not considered to be a specific area of interest unless it is important to understand driver behavior. In the same fashion, traffic management, vehicle maintenance, and cybersecurity are touched upon as related or supporting aspects rather than as independent areas of analysis.

The analysis does not restrict itself to specific behavioral tasks, sensing modalities, or modeling approaches, thereby addressing the limitations of previous reviews. This review consolidates a multitude of driver-behavior tasks and reviews the involved methods, data, evaluation measures, and supporting technologies. It also takes into account the cross-cutting needs, including real-time processing, preserving privacy, scalability, heterogeneous data, and generalization of models. This broader treatment helps identify gaps in integrating behavioral tasks and technologies and provides directions for developing more comprehensive driver-behavior analysis systems.

## 2 Background and scope of driver behavior analysis

### 2.1 Review methodology

Research on vehicular driving pattern analysis is reviewed in this manuscript as a narrative. To identify and synthesize pertinent studies with regard to key features of driver behavior, a structured literature-selection process was used, with the main areas of research selected being: driver behavior pattern recognition, detection of distracted driving, detection of abnormal driving behavior, driver profiling and identification, fuel-efficiency analysis, and driving-risk assessment. Studies related to sensor-based approaches, sensor fusion, emerging technologies, machine learning, and deep learning were also taken into consideration for driver behavior analysis.

The literature identification and selection process involved identification, screening, and assessment of eligibility and inclusion. During the identification stage, 698 records were considered, with 652 records identified via database searching and 46 identified from other sources. A total of 522 records after deduplication were screened using title and abstract. After the screening process, 236 full-text articles were evaluated for eligibility. Using the relevance and eligibility criteria, 117 studies were included for the final review. Figure 1 presents a complete overview of the study-selection process.

**Figure 1:**
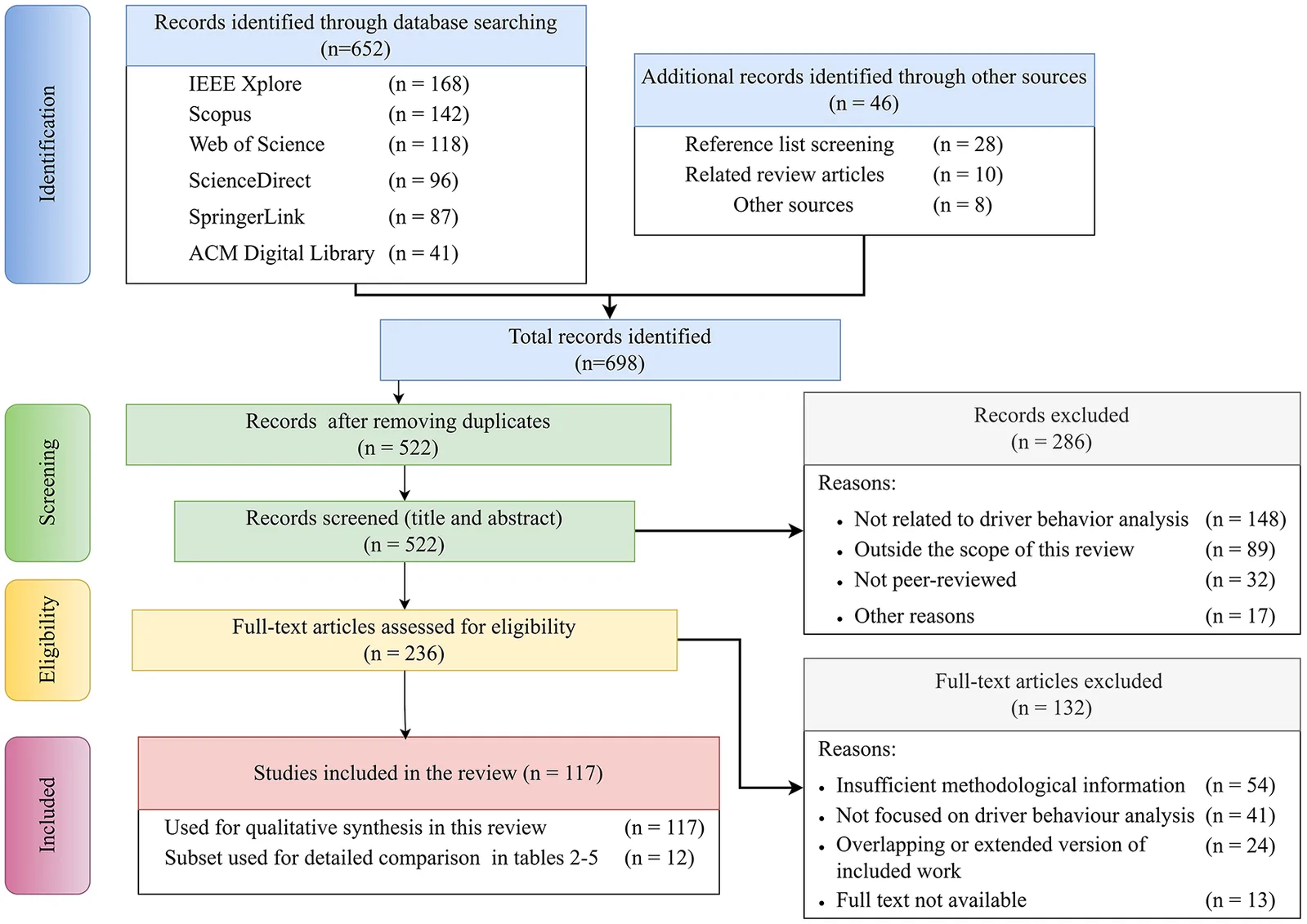
PRISMA-based flow diagram of the literature identification and study-selection process.

### 2.2 Core tasks in driver behavior analysis

The study of driver behavior is the cornerstone of comprehending how people drive cars, and the scope of such behavior is so large, including various vital dimensions like the control of speed, road etiquette, and adherence to traffic laws. Such a detailed study of drivers' behavior is helpful not only in the evaluation of road safety, but it can also be critical in determining the risks that might be lurking along the road. Questioning these behaviors provides better insight into driving habits, creating an environment to take better steps in road safety and prevent accidents. Fuel efficiency is also an aspect of road safety and efficient driving (Yaswanth and Babu, 2024). This task takes a systematic approach to grasping the efficiency of vehicle use of fuel resources. It examines several aspects, such as driving behavior, vehicle maintenance, and engine performance. With an increasingly fuel-efficient vehicle, the price savings are not the only benefits; the increased fuel efficiency provides a more sustainable method of transport, which in the long run will also lead to a less harmful environment and a green future. Driver cognitive distraction is one of the most important issues to identify and address within driver behavior analysis. This problem is concerned with calculating and correcting distractions that cause the mental focus of the driver to be redirected toward the primary task of driving (Shang et al., 2021). Such distractions may include texting and phone use, daydreaming, and all of them will be life-threatening to drivers on the road. Therefore, the most important thing to do is to deal with cognitive distractions to prevent accidents that emerge due to inattentiveness and divided attention. At the same time, another crucial task is detecting abnormal driving behavior. This project is aimed at detecting abnormal driving patterns or behaviors that can be considered as warning signs of impaired driving or unsafe road conditions. These patterns may be expressed in the form of abrupt accelerations, rough braking, or unpredictable lane changes. Early identification and action on such deviations from normal driving practices are crucial in preventing accidents and improving road safety in general. Driver behavior profiling is important in the wider driver behavior analysis framework. This entails the development of a detailed description of a driver's profile, in terms of habits and tendencies. Parameters, such as favorite routes, average speed, and driving time, are subject to examination. The experience of profiling leads to the individualization of driving recommendations and offers the framework on which the degree of risk associated with individual drivers can be evaluated. Another aspect of driver behavior assessment is driver steering behavior analysis. It entails a precise analysis of the steering inputs of a driver, such as the steering movement and corrections on a steering wheel. The given analysis gives a good understanding of the level of attentiveness and control of a vehicle on the part of the driver, which gives an insight into the capabilities and habits of the driver. Driver identification takes the front line in terms of controlling vehicle fleets and providing security. This is a task that is concerned with the verification of the person driving a vehicle. Proper identification is invaluable to proper fleet management, so that the appropriate individual is accountable for their actions on the road, and to observe data and interests of drivers in particular and their behavior. At the same time, the fight against distracted driving is getting more serious as it involves the challenge of detecting distracted driving. This is an essential exercise of finding cases of drivers getting distracted as a result of different activities like texting, eating, or using electronic gadgets while driving. These distracted driving behaviors can be identified and dealt with, and this is significant to improving road safety and reducing the number of accidents due to lapses in concentration; in the context of insurance and risk management, insurance risk pricing and claims forecasting are essential. Through data analysis, this activity evaluates the risk of insuring a certain driver. It entails forecasting insurance claims due to the behavior of the driver and other factors of interest so that insurance firms can accurately price their policies, take control of possible risk in advance, and ensure policyholders are fairly priced. Collectively, all these interrelated activities in the sphere of driver behavior study and road safety constitute a comprehensive system that would lead to improvement in driving patterns, fewer accidents, and safer and more sustainable use of the roads in the future.

Table 2 presents a brief description of several activities on driver behavior analysis and related issues, emphasizing the role played by various drivers (Vyas et al., 2021; Misra et al., 2023; Ma et al., 2023; Abdelrahman et al., 2020; Azadani and Boukerche, 2022; Chandra et al., 2021; Chen et al., 2023; Sun et al., 2023; Ping et al., 2023; McDonnell et al., 2023; Yang et al., 2023; Sethuraman et al., 2023) in the activities. Every row in the table represents a particular task, starting with profiling of driver behavior for fuel efficiency monitoring and all the way to insurance risk prediction. The existence of checkmarks (✓) in the columns that represent each driver means that they were involved in or relevant to the particular task.

**Table 2:** List of tasks to outline the driving patterns.

Published papers												
	Vyas et al. (2021)	Misra et al. (2023)	Ma et al. (2023)	Abdelrahman et al. (2020)	Azadani and Boukerche (2022)	Chandra et al. (2021)	Chen et al. (2023)	Sun et al. (2023)	Ping et al. (2023)	McDonnell et al. (2023)	Yang et al. (2023)	Sethuraman et al. (2023)
Tasks												
1. Driver behavior	✓	X	X	X	X	✓	✓	✓	X	X	✓	✓
2. Fuel efficiency	✓	X	X	X	X	X	X	X	X	X	X	X
3. Driver cognitive distraction	X	✓	X	X	X	X	X	X	X	X	X	X
4. Abnormal driving behavior	X	X	✓	X	X	X	X	X	X	X	X	X
5. Driver behavior profiling	X	X	X	✓	X	X	X	X	X	X	X	X
6. Driver steering behavior analysis	X	X	X	X	✓	X	X	X	X	X	X	X
7. Driver identification	X	X	X	X	✓	X	X	X	X	X	X	X
8. Distracted driving detection	X	X	X	X	X	X	X	X	✓	X	X	X
9. Insurance risk pricing and claims prediction	X	X	X	X	X	X	X	X	X	✓	X	X

The major task in the article (Vyas et al., 2021) is creating a predictive system of driving behavior and fuel efficiency. This entails behavioral modeling, score and fuel efficiency calculations, and optimization of trips depending on the driving patterns. There is also an interest in seeking practical applications of AI such as stress management among drivers and fleet support. The main aim of the article (Misra et al., 2023) was to find characteristics of various data sets in order to categorize distracted and non-distracted drivers across the cognitive distraction line. This consisted of data capture from a driving simulator study involving a range of driving situations and the adoption of classification algorithms such as Random Forest, Decision Trees, and Support Vector Machines. The research also sought to identify how road type influences the behavior of the drivers.

The Long Short-Term Memory Residual (LSTM-R) algorithm was introduced in order to detect various forms of abnormal driving behaviors in real time (Ma et al., 2023). They used a temporal window-based, sliding method using historical data in the fitting of an LSTM network to produce residuals in the diagnosis of continuous abnormal behavior. The sensitivity of the algorithms to the parameters, percentages of aberrant behavior, and their comparison with other algorithms were analyzed in the study to determine their effectiveness. The authors aimed to develop a powerful data-based approach to the assessment of the risk profile of drivers based on the data of the SHRP2 Naturalistic Driving Study (Abdelrahman et al., 2020). Some of the steps taken included the identification of behavioral risk predictors, creation of a feature matrix, sensitivity analysis, selection, configuration, and comparison of ML models to achieve the desired objective of predicting a risk probability, and finally, a discussion of how the proposed model can be integrated into a driver profiling system, which is to be hosted in the cloud.

The studies in Tables 2–5 were chosen because they are relevant to the scope of this study, they provide a contribution to the analysis of driver behavior, and because there is sufficient methodological and experimental information that can be used for comparative analysis. The selected studies that will be detailed and compared are those identified as references (Vyas et al., 2021; Misra et al., 2023; Ma et al., 2023; Abdelrahman et al., 2020; Azadani and Boukerche, 2022; Chandra et al., 2021; Chen et al., 2023; Sun et al., 2023; Ping et al., 2023; McDonnell et al., 2023; Yang et al., 2023; Sethuraman et al., 2023) and are analyzed in Tables 2–5 from various perspectives.

**Table 3:** List of methodologies to analyze the driving pattern.

Published papers												
	Vyas et al. (2021)	Misra et al. (2023)	Ma et al. (2023)	Abdelrahman et al. (2020)	Azadani and Boukerche (2022)	Chandra et al. (2021)	Chen et al. (2023)	Sun et al. (2023)	Ping et al. (2023)	McDonnell et al. (2023)	Yang et al. (2023)	Sethuraman et al. (2023)
Methods/models used												
1. Machine learning models (mmoe, moo, hmm)	✓	X	X	X	X	X	X	X	X	X	X	X
2. Machine learning models (rf, svm, dt, nb, knn)	X	✓	X	X	X	X	X	X	X	X	X	X
3. Lstm	X	X	✓	X	X	X	X	X	X	X	X	X
4. Machine learning: supervised learning	X	X	X	X	X	X	X	X	X	X	X	X
5. Machine learning models(dt, svm, dnn, elm, knn, rf)	X	X	X	✓	X	X	X	X	X	X	X	X
6. Convolutional neural networks	X	X	X	X	✓	X	✓	X	X	X	X	X
7. Multi-layer perceptron	X	X	X	X	X	✓	X	X	X	X	X	X
8. Transfer learning	X	X	X	X	X	X	✓	X	X	X	X	X
9. Hybrid adaptive anti-noise clustering algorithm (hadanoc)	X	X	X	X	X	X	X	✓	X	X	X	X
10. Heuristic self-learning labeling algorithm (hsl-svm/nn)	X	X	X	X	X	X	X	✓	X	X	X	X
11. Temporal–spatial double-line dl network	X	X	X	X	X	X	X	X	✓	X	X	X
12. Causal and-or graph (c-aog)	X	X	X	X	X	X	X	X	✓	X	X	X
13. Tabnet	X	X	X	X	X	X	X	X	X	✓	X	X
14. Bilinear full-scale residual Swin Transformer network (birswint)	X	X	X	X	X	X	X	X	X	X	✓	X
15. AdaBoost multi-class support vector machine (MSVM) with cat mouse optimizer (CMO) algorithm	X	X	X	X	X	X	X	X	X	X	X	✓

**Table 4:** List of input parameters for methodologies to outline driver behavior.

Published papers												
	Vyas et al. (2021)	Misra et al. (2023)	Ma et al. (2023)	Abdelrahman et al. (2020)	Azadani and Boukerche (2022)	Chandra et al. (2021)	Chen et al. (2023)	Sun et al. (2023)	Ping et al. (2023)	McDonnell et al. (2023)	Yang et al. (2023)	Sethuraman et al. (2023)
Input												
1. Uah-driveset	✓	X	X	X	X	X	X	X	X	X	X	X
2. Static dataset	X	✓	X	X	X	X	X	X	X	X	X	✓
3. Static video dataset	X	X	✓	X	X	X	X	X	X	X	X	X
4. Shrp2andnds data set	X	X	X	✓	X	✓	X	X	X	X	X	X
5. Vpalab dataset	X	X	X	X	✓	X	X	X	X	X	X	X
6. Vbox-iisx10 gps	X	X	X	X	X	X	✓	X	X	X	X	X
7. Fchev test bench	X	X	X	X	X	X	X	✓	X	X	X	X
8. Auc distracted driver (aucd2), State Farm distracted driver (sfd2), NTU distracted driver (ntud2)	X	X	X	X	X	X	X	X	✓	X	X	X
9. Synthetic telematics dataset	X	X	X	X	X	X	X	X	X	✓	X	X
10. State Farm dataset	X	X	X	X	X	X	X	X	X	X	✓	X

**Table 5:** List of output parameters from methodologies to outline the driving behavior.

Published papers												
	Vyas et al. (2021)	Misra et al. (2023)	Ma et al. (2023)	Abdelrahman et al. (2020)	Azadani and Boukerche (2022)	Chandra et al. (2021)	Chen et al. (2023)	Sun et al. (2023)	Ping et al. (2023)	McDonnell et al. (2023)	Yang et al. (2023)	Sethuraman et al. (2023)
Output												
1. Accuracy	X	✓	X	✓	X	✓	✓	✓	✓	X	✓	✓
2. Precision	✓	X	✓	X	✓	X	✓	✓	X	✓	X	✓
3. Recall	✓	X	✓	✓	✓	X	✓	X	X	✓	X	✓
4. F-measure	✓	X	✓	✓	✓	X	✓	X	X	✓	X	✓
5. Roc	X	X	X	✓	X	X	X	X	X	✓	X	X
6. Area under the curve (AUC)	✓	X	✓	✓	✓	X	X	X	X	✓	X	X

The primary aim of this research was to overcome this issue of driver identification and the problem of impostor recognition in terms of driving behavior analysis. This entailed the creation of a deep learning-based framework that identified unique steering patterns using a 30-s data window of driving, which was used to differentiate the drivers. Another strategy that was presented by the research was the use of the system architecture and Siamese networks to verify the driver and identify the impostor (Azadani and Boukerche, 2022). The proposed methodology was evaluated against the existing benchmarks and the baseline approach on a dataset of 95 drivers. In this study, the problem was to create a method that employs machine learning and graph-theoretic techniques to forecast human driver behaviors (Chandra et al., 2021) exclusively based on the observed vehicle trajectories. It aimed to imitate the manner in which human beings perceive driver actions on the road and to use the features derived from trajectories to create a computational map of vehicle trajectories and driver actions. Experiments were conducted to show the stability and usability of this methodology on diverse real-world traffic data collected in different locations around the world and in simulation settings.

The main activity of the research consisted of suggesting a workable approach to identifying driving patterns (Chen et al., 2023), especially in the case of heavy-duty freight trucks, whose data samples are traditionally rather small because of the high costs of data collection. This approach has selected five driving behavior patterns, which comprise acceleration, deceleration, turning, lane changing, and lane keeping. It did so by constructing a Convolutional Neural Network (CNN) and merging data from various sources, including online car-hailing drivers' facial expressions and natural driving GPS data. Once the CNN model had been trained on large volumes of data, the study performed the process of fine-tuning the parameters of the model, using smaller data sets of heavy-duty freight vehicles. This demonstrated that transfer learning is quite effective even when small samples are used. The primary aim of the research was to incorporate driving behavior (Sun et al., 2023) into an adaptive Deep Reinforcement Learning (DRL)-based Energy Management Strategy (EMS) of a three-source Fuel Cell Hybrid Electric Vehicle (FCHEV). The EMS must make energy sources efficient and durable. The study aimed to achieve the best power distribution through the combination of a hierarchical power division approach, behavior recognition motivation, and a similar consumption minimum approach.

The purpose of this study was to enhance the recognition of distracted driving behavior (Ping et al., 2023) based on a new methodology: a combination of a Temporal-Spatial double-line DL network (TSD-DLN) with a causal AND-OR graph (C-AOG). In recognizing distracted driving postures, the TSD-DLN was concerned with the combination of attention features represented by dynamic optical flow and spatial representations of the video frames. C-AOG contributed causal knowledge to improve recognition robustness, manage behavior state shifts, and solve occlusions. To compare the method with state-of-the-art DL methods, we used a self-collected dataset and public datasets. The technique proved to be more effective in unbroken recognition and robustness to less-than-perfect observation conditions. The major task was Insurance Risk Pricing and Claims Prediction (McDonnell et al., 2023), which aimed to analyze the model performance in terms of the results in forecasting the insurance claim based on the synthetic telematics dataset. The experiment consisted in fine-grained identification of 10 behaviors of drivers through a suggested BiRSwinT network to distinguish similar behaviors on the basis of local actions of body parts, which are subtle.

The problem focused on improving fine-grained driver behavior recognition (Yang et al., 2023) with a new model, BiRSwinT, designed to identify complex behavioral actions. It aimed to identify 10 different driver behaviors by considering global appearances as well as local body part activities. The main aim of this research was to come up with a proper anomaly detection mechanism for an Advanced Driver Assistance System through the application of an obligatory algorithm (Sethuraman et al., 2023). The activities consisted of processing CPS information to improve driving conditions, high-dimensional noisy data, data pre-processing, feature extraction, and the recognition of anomalies in driving behavior using the AdaBoostMSVM with the CMO algorithm.

### 2.3 Computational approaches and techniques

The methods and techniques of computation described in the table given are a holistic method of undertaking research or analysis in relation to different machine learning models and techniques, especially supervised learning and deep learning. The methodology involves implementing these research approaches and examining various organizations or topics referred to as (Vyas et al., 2021; Misra et al., 2023; Ma et al., 2023; Abdelrahman et al., 2020; Azadani and Boukerche, 2022; Chandra et al., 2021; Chen et al., 2023; Sun et al., 2023; Ping et al., 2023; McDonnell et al., 2023; Yang et al., 2023; Sethuraman et al., 2023). To derive insights on many properties of the entities under inquiry, machine learning, deep learning, and similar techniques are applied. This methodology centers on machine learning. These can be people, things, or anything that arouses interest. One of the important features is the application of machine learning models such as MMOE, MOO, HMM, Random Forest (RF), Support Vector Machines (SVM), Decision Trees (DT), Naive Bayes (NB), K-Nearest Neighbors (KNN), and others. These models are used to analyze and obtain meaningful patterns, relationships, or predictions in the context of the research. Furthermore, the approach introduces a concept of supervised learning that puts emphasis on the use of decision trees, support vector machines, deep neural networks (DNN), Extreme Learning Machines (ELM), and Random Forest to process and predict outcomes. Supervised learning techniques involve the use of labeled training data and are applied in classification or regression tasks. To conclude, this research methodology has highlighted the need to apply a variety of methodologies in order to address complex research questions and issues efficiently.

Table 3 provides a detailed overview of various methods and models applied in several published articles on driver behavior studies and related areas. It outlines some of the methodologies utilized in these studies, as indicated by the methods/models in the first column. The existence of the checkmarks (✓) in the assigned columns, respectively, for each of the papers (Vyas et al., 2021; Misra et al., 2023; Ma et al., 2023; Abdelrahman et al., 2020; Azadani and Boukerche, 2022; Chandra et al., 2021; Chen et al., 2023; Sun et al., 2023; Ping et al., 2023; McDonnell et al., 2023; Yang et al., 2023; Sethuraman et al., 2023), indicates the use or implementation of a particular method/model in the context of a particular paper. This table will be a quick reference about the adoption of distinct methodologies in various research publications and allow one to simply see which approaches are used by the specified paper in the field of driver behavior analysis and associated tasks.

In order to fulfill the above tasks (Vyas et al., 2021), the system will forecast driver behavior with the help of MMoE based on past driving data and determine scores on behavior and fuel efficiency with the help of HMM. Trip recommendations will be done through multi-objective optimization, where behavior and fuel efficiency are the major considerations. Further, features were derived from the eye-tracking, physiological, and vehicle kinematics data to meet the task objectives (Misra et al., 2023). Differences in machine learning methods were adopted and compared to categorize driver cognitive distraction. The experiment entailed the young drivers undertaking tasks in a driving simulator of various road types, with and without auditory distractions, which were controlled. It was centered on establishing predictive characteristics of distraction classification and the effect of road types and their influence on driver behavior. Combining these strategies, the study hopes to come up with a holistic model not only for predicting and estimating fuel efficiency but also for factoring in cognitive distraction, the type of road, and auditory distractions, which will eventually add up to a more holistic explanation of vehicular driving behavior and safety factors.

The study has utilized non-intrusive data on smartphones to evaluate a variety of aberrant driving behaviors across Nanjing, China. When compared to AdaCost, SMOTEBoost, EasyEnsemble, LightGBM-residual, and linear regression residuals, the LSTM-R (Ma et al., 2023) algorithm was used to determine the effectiveness of the detection. The assessments included sensitivity analysis of parameters, optimization of the structure, and how proportions of abnormal behavior affected the detection performance. The study used a set of machine learning algorithms to predict the probability of risks and calculate their efficacy. Some of the algorithms employed included Machine learning models like Random Forest (RF), Decision Trees, Logistic Regression, Gradient Boosting, and Neural Networks (Abdelrahman et al., 2020). The framework selected, customized, and compared these algorithms to determine the best model for predicting risks. The assessment consisted of evaluating the performance of each algorithm with regard to the accuracy of its modeling and prediction of the risk profile of drivers based on the dataset. By doing sensitivity analysis and comparison, the research has identified the best algorithm to use in predicting risks, outlining its high ability in skewed-data prediction and capability to predict the probability of risk, especially with less driving time, as demonstrated by the Random Forest-based predictor.

The research focused on creating a deep learning-based system architecture (Azadani and Boukerche, 2022) oriented toward driving behavior analysis, specifically for driver identification and verification problems. The method was to apply one-dimensional temporal convolutions to extract distinctive information in the driving data, avoiding the need to perform much pre-processing and reducing computational complexity compared to recurrent neural architectures. Siamese networks were also used to generate driver encodings that can be verified and used to detect impostors, and to solve cases of few driving experiences per driver.

The approach that will be used in the research is probably the use of Convolutional Neural Networks (CNNs) (Chandra et al., 2021) in the machine learning platform to run and obtain characteristics of vehicle paths. CNNs are also able to capture sequential and spatial patterns, which means that they can be used with sequential data such as trajectory sequences. Such networks may be used to acquire hierarchical representations of the paths, discovering complex relations or patterns between space in the sequences of movements of cars. This method enables effective encoding of the trajectory data by the CNNs, which can then extract discriminative features for different behaviors of the drivers, which could improve the capability of the system in mapping the vehicle trajectories to human driver behaviors.

The study used a Convolutional Neural Network (CNN), along with data fusion from other sources such as real GPS data and driver faces, to identify specific driving-behavior patterns (Chen et al., 2023). The experiment demonstrated that transfer learning can be used to enhance the performance of recognition in such situations by fine-tuning the parameters of a pre-trained convolutional neural network (CNN) with a small set of heavy-duty freight vehicles. Based on this approach, an Energy Management Strategy (EMS) of a Fuel Cell Hybrid Electric Vehicle (FCHEV), which runs on adaptive Deep Reinforcement Learning (DRL), has been developed (Sun et al., 2023). It began with a hierarchical method of splitting power, which employed an adaptive fuzzy filter to establish the optimal power distribution among the different sources. A high-performance driving behavior recognition system was integrated to use graph-based methods to extract driving data features. The minimum principle introduced by Pontryagin considered optimal equivalents of different behaviors. The main idea was a sophisticated DRA, P-DQL, which included action trimming, adaptive learning, and space division mechanisms to improve decision-making. This algorithm combined the driving behavior-aware adaptive equivalent consumption minimization strategy (A-ECMS), balancing global learning and real-time implementation to optimize power allocation, which should reinforce energy efficiency and increase the power source lifespan. They decided to make use of the A-ECMS strategy, which is a combination of global learning and real-time implementation to improve power distribution, increase efficiency, and increase power source lifespan.

The main approach was to build a TSD-DLN to check distracted driving (Ping et al., 2023), which uses the temporal-spatial dual line structure to identify the distribution of attention and combine it with spatial features to identify the behavior. Moreover, a causal AND-OR graph (C-AOG) was incorporated to improve the inferential ability of the model, in that cause-and-effect relationships were established between the image semantics and flow of behavior. This combination enhanced detection of distracted driving as opposed to the conventional image-only DL approaches. The C-AOG-based inference model particularly overcame the problem of image noise and body agent concealment, and it exhibited high recognition accuracy. The output showed high recognition accuracy and AUC values, with little delineation of behavioral boundaries. In the study (McDonnell et al., 2023), TabNet, XGBoost, Logistic Regression, LightGBM, and NNs were used to estimate insurance claims on a telemetric dataset. The authors used performance metrics and model interpretability to compare each model.

The methodology uses an algorithm based on the BiRSwinT model, which aims to recognize fine-grained driver behavior (Yang et al., 2023). It starts with a dual-stream architecture that involves a transformer-based network and has two parallel streams, with each stream focused on addressing different dimensions of driver behavior. The global representation branch is used to capture the shape and appearance of the whole body, whereas the local residual branch uses the full-scale skip connections to identify minute actions of the local body parts in the behaviors. This arrangement allows the model to learn and extract multi-scale cues in driver actions. Then, a bilinear fusion strategy is proposed, planned with the help of a three-stage training plan. This plan is a gradual convergence of the global representation and the local detail features, acquired through the dual stream structure. In this amalgamation process, the model is able to leverage the synergy of the global as well as local information in the ultimate resultant higher recognition accuracy of various driver behaviors. The solution used AdaBoost MSVM with the CMO algorithm to detect anomalies in ADAS (Sethuraman et al., 2023). The proposed algorithm for ADAS anomaly detection utilized both the CMO algorithm and the AdaBoost MSVM. This method was created to address MSVM problems, including instability, overfitting, and poor performance. After optimizing the MSVM parameters with the CMO technique, we trained the model using power supply voltage data from the Jiangxi Bus Company.

### 2.4 Input

Table 4 provides a synopsis of the datasets that were used in different published papers (Vyas et al., 2021; Misra et al., 2023; Ma et al., 2023; Abdelrahman et al., 2020; Azadani and Boukerche, 2022; Chandra et al., 2021; Chen et al., 2023; Sun et al., 2023; Ping et al., 2023; McDonnell et al., 2023; Yang et al., 2023; Sethuraman et al., 2023). Every row defines a particular dataset that was applied in the respective research. The existence of a checkmark (✓) in the columns of each paper depicts the use or applicability of the corresponding dataset to the corresponding publication. These datasets include various sources; among them are UAH-DRIVESET, SHRP2&NDS, VPALAB, and various driver behavior datasets, such as AUCD2, SFD2, NTUD2, among others. Each dataset is a pioneering research endeavor that offers distinct insights into the study of driver behavior, non-dynamics analysis, and studies focused on telematics.

The system in article (Vyas et al., 2021) is based on historical driving data, including behavior data, fuel efficiency history, trip history, and possibly other data like ratings and driving experience, which is obtained by accessing datasets such as the UAH-DriveSet or other repositories. The data for this study were comprised of the data gathered (Misra et al., 2023) on 40 drivers in a driving simulator, which included eye-tracking, physiological, and vehicle kinematics data. The information contained in the dataset was related to different driving situations, road conditions, and distractions, such as controlled auditory communication tasks, and this presents a resourceful collection of varied and all-inclusive data to develop and test the proposed system.

The inputs consisted of kinematic information of the vehicle obtained in an experiment in Nanjing, China, with the help of a smartphone application (Ma et al., 2023). To train and test the model, we recorded individually by hand the occurrence of different anomalous driving behaviors and tested them against other algorithms. The model building and evaluation inputs were based on the SHRP2 Naturalistic Driving Study data (Abdelrahman et al., 2020), which provided a wide array of behavioral risk predictors and events reflecting crash and near-crash conditions and balanced baseline driving conditions.

The data used in this research were the driving behavior data obtained from 95 drivers (Azadani and Boukerche, 2022), which were oriented toward steering behavior analysis in the 30-s driving data. This dataset was used to train and test the proposed deep learning-based system in the driver identification, verification, and impostor detection tasks. The data used in this research were vehicle trajectory data gathered in real-life traffic data in different parts of the world such as the U.S., India, China, and Singapore, and simulation data (Chandra et al., 2021). These trajectories were used to obtain the features of driver behavior and to train the machine learning model to build mappings between the trajectories and behaviors of human drivers.

Among various sources of data were natural driving GPS and face expressions of drivers that were collected and accessed online through car-hailing services (Chen et al., 2023). The CNN model was trained and fine-tuned on these sources of data to identify five patterns of driving behavior. The inputs into the study were real-world data of the FCHEV test bench (Sun et al., 2023) and mathematical modeling of the power sources in the vehicle. Furthermore, data on driving behavior and other driving cycles were used to simulate and test the proposed EMS.

The research utilized a mix of databases, with one of them being self-collected (Ping et al., 2023) and two publicly accessible ones, namely, AUC distracted driver (AUCD2) and State Farm distracted driver (SFD2). NTU distracted driver (NTUD2) is the self-collected data, which was utilized to specifically evaluate the efficacy of the suggested approach to the recognition of distracted driving. The study sought to prove that the superiority of the proposed approach in detecting distracted driving behaviors could be demonstrated by comparing and evaluating it against these datasets. The input data included a synthetic telematics dataset (McDonnell et al., 2023), in which there was relevant information about the drivers' behavior, claims, and policyholders' data, all of which apply to insurance risk assessment and claim prediction.

The proposed fine-grained driver behavior recognition system, BiRSwinT, used input data that were sequences of the State Farm Distracted Driver data (Yang et al., 2023). This data set was inclusive of a number of different cases representing a range of driver behaviors, in particular, 10 different behaviors related to distracted driving. It had a set of pictures of different actions and behaviors of drivers when involving themselves in various distractions, which was a detailed account of the minor yet significant aspects in the actions of drivers. The abundance of information in the dataset helped the model to identify local body part behavior complexities in these behaviors, and a robust and detailed analysis was done to accurately identify the behavior. The experimental data assumed in this research paper included data on power supply voltage used in the process of driving vehicles by the Jiangxi company (Sethuraman et al., 2023). The information available in this dataset was necessary for identifying anomalies in driving behaviors, which is important in establishing normal and unusual driving events.

### 2.5 Evaluation metrics

The data presented in Table 5 provide the statistics of the output of other published articles (Vyas et al., 2021; Misra et al., 2023; Ma et al., 2023; Abdelrahman et al., 2020; Azadani and Boukerche, 2022; Chandra et al., 2021; Chen et al., 2023; Sun et al., 2023; Ping et al., 2023; McDonnell et al., 2023; Yang et al., 2023; Sethuraman et al., 2023). They consider the key performance indicators used to assess the various models or approaches used in these papers. Metrics such as Accuracy, Precision, Recall, F-Measure, ROC (Receiver Operating Characteristic), and AUC (Area Under the Curve) are important measures for evaluating the effectiveness of the methodologies in the studies in question. The availability of checkmarks (✓) shows the inclusion or the reporting of these metrics in the output assessment of the cited papers.

In article (Vyas et al., 2021), the system output parameters include Precision, Recall, F-measure, and Area Under the Curve (AUC). Precision is a measure of accurate positive prediction, Recall is a measure of true positive rate, F-Measure is a measure of balancing Precision and Recall, and AUC is found as an overall measure of the model performance. The system should have high Precision (positive predictions that are accurate) and Recall (true positive rate) with a balanced F-Measure and with a large AUC, which means that the model will perform well when recommending trips and predicting behavior. The results of the study (Misra et al., 2023) saw the identification of the main features, including the area of the pupil and its vertical and horizontal movement, as predictors of driver distraction, and the classification accuracy of which introduced an average of 90% in various types of roads. Also, some understanding of the effect that types of roads have on driver behavior was obtained, which can be applied in designing distraction monitoring systems in practice. These results indicate that the system can provide important information that can be used to improve the safety of the driver and the driving experience.

Precision, Recall, F-Measure, and Area under the Curve (AUC) (Ma et al., 2023) were the output parameters used to measure the performance of the algorithm. Precision was used to measure how accurate positive predictions were, Recall was used to measure the true positive rate, F-Measure was used to balance Precision and Recall, and AUC was used to measure the overall performance of the detection algorithm. The LSTM-R algorithm is observed to be effective as it has high Precision, Recall, and F-Measure, with the maximum F1-score of 0.866, and was found to perform better than other algorithms, indicating that it is very effective at detecting abnormal driving behaviors in real-time. The output parameters were used to evaluate the performance of the framework to measure the Accuracy, Recall, F-measure, Receiver Operating Characteristic (ROC) curve, and Area Under the Curve (AUC) (Abdelrahman et al., 2020). Precision was an overall measure of accuracy, Recall was a measure of the true positive rate, F-Measure was a measure of balancing Precision and Recall, and ROC and AUC were indications of the discriminatory ability of the model. The experiment showed a high level of Accuracy, Recall, and F-measure of the machine learning models, and the predictor based on the Random Forest performed better. The ROC curve and AUC analyses attested to the strength of the structural framework in ensuring effective identification of the risk profile of drivers, thus highlighting its possible use in driver profiling services in car insurance telematics and fleet management sectors.

According to the study, the output parameters were Precision, Recall, F-Measure, and Area Under the Curve (AUC) to find out the performance of the proposed system in driver identification, verification, and impostor detection exercises (Azadani and Boukerche, 2022). Precision and Recall are measures of the accuracy of positive predictions and the rate of true positives, respectively; F-measure was a balance between Precision and Recall, and AUC was the performance of the model. The methodology had high Precision, Recall, and F-Measure that exceeded current benchmarks in accuracy in driver identification in top-1 accuracy, top-3 accuracy, and top-5 accuracy. Moreover, the efficiency of the system in terms of driver authentication, especially with a small number of driving incidences per driver, was also evident. The extension using this method to detect impostors was also found to have better performance than replicated benchmark algorithms, which suggests the strength of the proposed system. Accuracy (Chandra et al., 2021) was the output parameter of the study used to measure the performance of the approach in modeling human driver behaviors on the basis of the observed vehicle movements. The objective of the methodology was to forecast driver behaviors only based on trajectory data with the help of graph-theoretic tools and machine learning. The conclusion identified the strength and overall applicability of the approach to a variety of real-world traffic data sets and simulations and hailed its potential in behaviorally-directed autonomous navigation. Precision was used as an evaluation of how effective the method was in predicting human driver behaviors accurately, which shows that it is reliable in modeling real-time driver behaviors using vehicle trajectories only.

Accuracy, Precision, Recall, and F-Measure (Chen et al., 2023) were the output parameters of the study that evaluated the effectiveness of the suggested methodology in identifying the driving behavior patterns. With a Precision value of 0.82 on average, Recall of 0.87, and F1 Score of 0.84, the CNN model has a score of 0.824 in the field of online car-hailing, which demonstrates its effectiveness in identifying the different driving behavioral patterns with an adequate degree of correctness. Also, the recognition performance enhanced considerably through transfer learning, providing an accuracy value of 0.80 with a significant improvement in Precision, Recall, and F-Measure, indicating the strength of the method and its effectiveness, particularly in a case where data samples are low. The output parameters of the study were aimed at the assessment of the suggested adaptive Deep Reinforcement Learning (DRL)-based Energy Management Strategy (EMS) of a three-power-source Fuel Cell Hybrid Electric Vehicle (FCHEV). The suggested technique, dubbed P-DQL, exhibited excellent performance in simulations, with an average of 49.9% drop in hydrogen use in contrast to the conventional ECMS technique (Sun et al., 2023). It also reduced overall usage cost by 31.4%. These results represent the outstanding promise of the given EMS in terms of fuel economy and decreasing hydrogen usage, its capacity to optimize power distribution, and increasing the efficiency of FC and battery systems in the FCHEV. Nevertheless, the research did not mention accuracy and precision as specific evaluation measures for the proposed EMS.

The accuracy of the proposed method was greatly enhanced compared to the state-of-the-art DL methods (Ping et al., 2023). The combination of Temporal -Spatial double-line DL network (TSD-DLN) and causal AND-OR graph (C-AOG) greatly boosted distracted driving behavior recognition. The precision of the technique was greater than that of other DL models in dealing with sequential frames. The experiment results on the public and self-collected datasets were always characterized by a higher accuracy in acquiring the distracted driving behavior. The fusion model in the PTD-D dataset attained an average accuracy of 0.875 and an AUC of 0.883, which is higher than the traditional DL approaches that use fixed image recognition. The method demonstrated a strong ability to provide proper continuous recognition even in cases of incomplete observation, which is an indication of its possibility of use in the real world. The study found that these models made correct predictions of such claims in the telematics dataset (McDonnell et al., 2023). It showed greater accuracy, precision, recall, and F1-scores in capturing sparse claims data. Furthermore, TabNet also produced results that could be interpreted easily and was able to pinpoint major trends in the telematics and policyholder information that can be useful to insurers. However, it was more difficult to optimize hyperparameters and took longer to train than alternative models such as XGBoost.

The results were the recognition accuracy of the suggested BiRSwinT model, compared to the AUC V1 and V2 datasets, with Top-1 accuracies of 93.235% and 92.253, respectively (Yang et al., 2023). Moreover, the generalization experiments based on the State Farm data proved the excellent functioning of the model, as the Top-1 accuracy rate was achieved at 83.335. The findings indicated that the suggested AdaBoost MSVM with CMO algorithm was better than other current algorithms with regard to precision, recall, accuracy, and F-score (Sethuraman et al., 2023). The model had an accuracy of 91.45%, F-score of 94.45%, precision of 93.89%, and a recall of 94.90%. Moreover, the method demonstrated better results in detecting anomalies that are connected to DoS, R2L, Probe, and U2R attacks than RAD-EI, NSAD, NSGA-II, and ECBD methods.

A detailed comparison of the selected studies (Vyas et al., 2021; Misra et al., 2023; Ma et al., 2023; Abdelrahman et al., 2020; Azadani and Boukerche, 2022; Chandra et al., 2021; Chen et al., 2023; Sun et al., 2023; Ping et al., 2023; McDonnell et al., 2023; Yang et al., 2023; Sethuraman et al., 2023) according to research tasks, datasets, methodology, evaluation measures, main results, strengths, and limitations is introduced in Table 6. The results of the comparison demonstrate that the studies focus on particular aspects of driver behavior analysis with machine learning, deep learning, transfer learning, and optimization-based approaches. Study characteristics are also varied (data sets, experimental conditions, evaluation measures). Such differences reveal the existing diversity of approaches and give insight into limitations regarding the diversity of the datasets, real-world validation, generalization, and integration of several driver behavior tasks.

**Table 6:** Comprehensive comparison of selected studies on driver behavior analysis.

Authors and Year	Task	Dataset	Model/method	Evaluation method	Main results	Strengths	Limitations
Vyas et al. (2021)	Driver behavior prediction and fuel-efficiency analysis	UAH-DriveSet; 6 drivers; 500 min of driving data	MMoE, HMM, and Multi-Objective Optimization (MOO)	Accuracy, AUC, Precision, Recall, F-measure, MSE, R^2^	The accuracies were: 95% for driver behavior prediction and 0.0101 MSE and 0.7567 R^2^ for fuel-efficiency estimation using Linear Regression.	Combines driver behavior, fuel efficiency, and trip recommendation in a single approach.	The number of drivers in the dataset is very small, which limits the diversity of driver characteristics.
Misra et al. (2023)	Cognitive distraction detection	Driving simulator dataset; 40 drivers, 14,076 data points; six road scenarios	Random Forest, Decision Tree, SVM using eye-tracking, physiological, and vehicle-kinematic features	Accuracy; 10-fold cross-validation	The average accuracy for the reduced eye-feature set was 90% for all road types.	Combines three data modalities and examines the effect of different road types.	The study was conducted with a driving simulator and restricted to young drivers (18–23 years).
Ma et al. (2023)	Real-time abnormal driving behavior detection	Smartphone-based vehicle-kinematic data; Nanjing, China; 47,100 samples	LSTM-Residual (LSTM-R) with time-window residual detection	Precision, Recall, F1-score, ROC/AUC	The maximum value of the F1-score = 0.866, and F1-scores were nearly maintained in the normal range when 10% abnormal data was applied for training.	Identifies continuous abnormal behavior and minimizes reliance on large volumes of abnormal labeled data.	This experiment featured only one test route and one expert driver, which reduced diversity and generalization.
Abdelrahman et al. (2020)	Driver behavior profiling and risk prediction	SHRP2 NDS; 3,542 drivers; more than 35 million miles	Supervised ML: DT, SVM, DNN, ELM, KNN, RF	Accuracy, precision, recall, F1-score, ROC/AUC; 10-fold cross-validation	RF's results for the general splitting approach were 87% accuracy, an F1-score of 0.93, and average precision of approximately 97% under the general splitting approach and average precision of about 90% under cross-validation.	Compares multiple supervised ML models on a large, naturalistic driving dataset.	The performances rely on the naturalistic driving events and behavioral risk predictors that are available.
Azadani and Boukerche (2022)	Driver identification, verification, and impostor detection	VPALAB naturalistic dataset; 95 drivers; Istanbul, Turkey	Temporal Convolutional Network and Siamese-network-based architecture	Top-1/Top-k accuracy, precision, recall, F1-score, AUC, EER; cross-validation	The Top-1 accuracy was near 90.4% for 2 drivers and 25.8% for 60 drivers, while the verification AUC and EER were 83.30% and 24.7%, respectively.	Captures temporal steering characteristics, capable of both identification and impostor detection using short driving windows.	There is a trade-off for identification accuracy when there are increasing numbers of drivers, and also for the number of driver-behavior representations to be used for impostor detection.
Chandra et al. (2021)	Human driver behavior prediction	Real-world trajectory datasets from the U.S., India, China, and Singapore, plus simulation	Graph-theoretic feature extraction, StylePredict, MLP, and GCN-based navigation	Time Deviation Error (TDE), weighted classification accuracy, simulation-based evaluation	The evaluated driving styles achieved an average TDE of less than 1 second, compared to existing approaches, with improvement by up to 25%.	Takes traffic information from geographically distributed traffic and explicitly models the interactions between vehicles.	The authors conclude that the current method can only be applied to straight roads and needs to be extended to intersections, roundabouts, and merging situations.
Chen et al. (2023)	Driving behavior recognition across vehicle types	Online car-hailing and heavy-duty freight-vehicle driving data	CNN with multi-source data fusion and transfer learning	Accuracy, precision, recall, F1-score, ROC/AUC	The accuracy obtained by transfer learning was 0.80, and 0.64 without transfer learning.	Incorporates driver facial-expression information and vehicle kinematics for handling limited training data for heavy-duty vehicles.	Recognition performance relies on the transfer between source vehicle domain and target vehicle domain.
Sun et al. (2023)	Driving-behavior recognition and energy management for FCHEV	FCHEV test bench; standard and extreme driving cycles including FTP75, NYCC, NEDC, HWFET, WLTC, US06, and SC03	HADANOC + HSL-SVM/NN; adaptive soft DRL-based P-DQL energy management	Accuracy, precision, recall, specificity; hydrogen consumption and total cost	The overall performance of the driving-behavior recognizer was 95.8%, while the whole driving cycle was 98.5% accuracy, 92.1% precision, 95.0% recall, and 97.5% specificity. P-DQL saved 49.9% in hydrogen consumption and 31.4% in total cost compared to ECMS.	Directly links driving behavior to energy-management decisions and fuel economy.	The energy-management evaluation is mainly simulation/test-bench oriented and is not based on large-scale real-road validation.
Ping et al. (2023)	Distracted driving behavior recognition	AUC Distracted Driver (AUCD2), State Farm (SFD2), and NTU Distracted Driver (NTUD2) datasets; plus STD, PTD, and PTD-D test datasets	Temporal-Spatial Double-Line DL Network (TSD-DLN) + Causal And-Or Graph (C-AOG)	Average Accuracy, F1-score, ROC/AUC	The fusion model on PTD-D got an average accuracy of 0.875 and AUC of 0.883, and on PTD it got an average accuracy of 0.895 and F1 score of 0.863.	Incorporates temporal, spatial, and causal information, and increases noise and occlusion robustness.	The authors indicate that the difference in noise intensity and duration was not investigated in detail.
McDonnell et al. (2023)	Insurance risk pricing and claims prediction	Synthetic telematics dataset; 100,000 samples and 52 variables	TabNet; comparison with XGBoost and Logistic Regression	F1-score, precision, recall, AUC, ROC, Matthews Correlation Coefficient	TabNet was the most accurate, with the highest F1 score of the three testing levels and was more accurate in recall in two of the testing levels; TabNet also provided global and local interpretability.	Integrates the power of predictive modeling with the interpretability of models for insurance use in the telematics domain.	The dataset was synthetic, and TabNet also demands a significant amount of training and hyperparameter tuning.
Yang et al. (2023)	Fine-grained driver behavior recognition	AUC V1, AUC V2, and State Farm datasets	BiRSwinT: bilinear full-scale residual Swin Transformer	Top-1, Top-3, Top-5 accuracy; precision, recall, F1-score; generalization evaluation	Top-1 accuracy of 93.235% on AUC V1 and 92.253% on AUC V2; C0–C8 accuracy reached 95.127% on AUC V2.	Captures both global driver appearance and subtle local actions via dual-stream representation and bilinear fusion.	Visually similar behaviors, especially in category C9, are hard to distinguish with fine-grained recognition.
Sethuraman et al. (2023)	Normal/abnormal driving behavior monitoring for ADAS	Jiangxi Bus Company power-supply voltage data; 17,000 records from 50 sensors	AdaBoost Multi-class SVM optimized using Cat Mouse Optimizer (CMO)	Accuracy, precision, recall, F-score, Friedman test, *post-hoc* Nemenyi test	Achieved 91.45% accuracy, 93.89% precision, 94.90% recall, and 94.45% F-score.	Cleanses noisy and high-dimensional ADAS data with feature reduction and parameter optimization.	Power-supply sensor data from a bus system is used for evaluation, which restricts the variety of vehicle types and sensing modalities.

## 3 Foundations of driver behavior modeling

### 3.1 Evolution of driver behavior research

#### 3.1.1 Early Observational Studies (Pre-2000s)

The initial research to study driver behavior used observational research methods, as academics cataloged driving habits and behaviors by handwritten records of driving in a controlled setting or in the real world. Initial studies were based on basic human factors, which included reaction time, attention span, decision-making, and cognitive load in the process of driving (Al-Sultan et al., 2013). The use of psychological research was critical in comprehending the way in which stress, exhaustion, and distractions affected driving performance. Questionnaires, driving simulators and on-road experiments were frequently employed, but these methods were typically narrow in scope, prone to observer bias and could not be scaled.

#### 3.1.2 Introduction of telematics and sensors (2000s)

The introduction of telematics and in-vehicle sensors revolutionized the research on driver behavior because they allowed collecting data in real-time and continuously. Superior sensors are integrated into cars to check the rate, acceleration, deceleration, lane keeping, and steering movements (Young et al., 2020). The creation of Naturalistic Driving Studies (NDS) was a significant change, as it has enabled researchers to obtain significantly large volumes of data related to real-life driving conditions without affecting the natural behavior of the driver. Technologies like GPS, dash cameras, and biometric sensors became a part of the research about driving habits, road interactions, and risk factors (Reicherts et al., 2021). This was also the era of the emergence of the black-box insurance models, where the insurers were able to use telematics data to determine individual behavior on the road.

#### 3.1.3 Integration of AI/ML (2010s Present)

Due to the development of artificial intelligence (AI) and machine learning (ML), driver behavior analysis shifted from simple descriptive research to multifaceted predictive models. The classification of driving styles, the identification of unsafe behaviors (e.g., drowsy driving, distraction, or aggressive acceleration), and the feedback tailored to the specific person were made possible by the use of machine learning algorithms, which helped to ensure safety on the road. The methods of computer vision and deep learning were used to process facial expression, gaze direction, and hand signals to identify driver fatigue or inattentiveness (Tong et al., 2019). AI-based systems also improved the Advanced Driver Assistance Systems (ADAS), where real-time interventions could be used to avoid accidents.

#### 3.1.4 Connected and autonomous systems (present and future)

Recent developments in driver behavior analysis include connected-vehicle technologies and autonomous driving systems (Nascimento et al., 2019). Vehicle-to-Everything (V2X) communication enables cars to communicate with each other, infrastructure, and cloud systems and, as a result, AI-based models can anticipate the intent of the driver and adapt to the current traffic conditions. Autonomous vehicles enable the use of deep learning and sensor fusion to optimize behavioral models and the provision of digital twins and metaverse simulations as a means to provide large-scale virtual testing platforms. Future research is expected to explore federated learning for privacy-preserving behavioral modeling, blockchain for secure data sharing, and artificial intelligence for safer and more effective driving functions.

### 3.2 Role and importance of AI/ML in driver behavior

Machine learning (ML) and artificial intelligence (AI) are key factors in understanding driver behavior and facilitating real-time monitoring. The technologies deliver data from various vehicle sensors as well as telematics, biometric, and external conditions, and from this data they can detect driving behavior. AI models can help identify risky behaviors like sudden braking and sharp lane changes, as well as inattention due to fatigue, to improve road safety. There is also the addition of AI that will increase risk assessment in usage-based insurance by assessing personal driving styles and changing the payments. ML algorithms can be used to improve the performance of drivers, fuel consumption, and maintenance planning in fleet management to operate in a cost-effective and safer way. In addition to the aspects of safety and efficiency, AI and ML are part of the technologies of autonomous and assisted driving (Al Asmari et al., 2025). Another field of application of AI is Advanced Driver Assistance Systems (ADAS), which process real-time road conditions, identify obstacles, and automate emergency responses. The driving experience is further personalized by AI-driven personalization that modifies the vehicle settings and preferences in the navigation, considering the behavior of the driver. Nevertheless, the problems of data privacy, algorithmic discrimination, and the threat of cybersecurity need to be addressed to make the implementation ethical and secure. With the further development of AI-driven analytics, it will be integrated with connected cars and intelligent infrastructure, making the driving experience safer, more efficient, and sustainable.

### 3.3 Influencing factors in driver behavior

The interaction between cognitive, technological, and environmental factors that influence driving decisions and outcomes plays a dynamic role in driver behavior. Stress, fatigue, and distraction are all cognitive and emotional states that can affect decision-making, but AI-driven systems can now analyze facial recognition, voice analysis, and other physiological sensors to detect these conditions and begin to instigate adaptive changes. The sensor-based monitoring, which operates with LiDAR, infrared cameras, gyroscopes, and biometric inputs, allows vehicles to recognize hazards in real-time, yet this method encounters the issues of price, consistency of data, and the requirement of computation (Yang et al., 2025). Vehicle-to-Everything (V2X) connection is more situational awareness-enhancing because it enables the vehicle to share traffic and hazard information, but it has cybersecurity and latency issues. The next level of reduction of human error is provided by advanced driver assistance systems (ADAS) and autonomous technologies, which handle such tasks as lane maintenance and emergency braking, but which demand constant updates to the software and precise mapping of the environment. With the increased use of such smart systems, the importance of safe and transparent access to sensitive information about the driver becomes highly important, which leads to the introduction of blockchain and the use of decentralized storage to increase trust. Moreover, the driving adaptation that is socially powered by AI will allow improving safety and comfort by personalizing vehicle settings and interventions, depending on the preferences and behavioral patterns. Outside factors such as weather, the quality of the road surface, and the density of traffic also have a significant impact on driver responses, and AI systems are becoming more popular in modulating vehicle dynamics based on the dynamically changing external conditions. The behavior can be modified with social and behavioral factors, including peer comparison, gamification incentives, and in-vehicle interaction, whereas the risk anticipation tools that are powered by AI should anticipate potential dangers and prescribe the necessary correction steps before the event takes place (Devarajan et al., 2025). Last but not least, environmental considerations, energy efficiency, and eco-driving are also becoming a prerequisite, with AI systems optimizing fuel and promoting environmentally friendly driving behaviors through real-time fuel consumption feedback and route planning. All these intertwined variables form the contemporary picture of driver behavior analysis and need a combined AI-based system to enhance safety, performance, and sustainability. The major factors affecting driver behavior, as mentioned, are human factors, vehicle factors, road factors, environmental factors, and traffic factors, which can be seen in Figure 2. Driver characteristics, including driving experience, cognitive state, attention, and driving style, interact with vehicle condition, road characteristics, weather and traffic conditions, and the driving environment (Kochetova, 2022). These interacting factors can affect driver perception, decision-making, workload, and resulting driving behavior. Thus, both driver-specific characteristics and the driving environment should be taken into account when analyzing driver behavior (Tinella et al., 2020; Di Flumeri et al., 2018).

**Figure 2:**
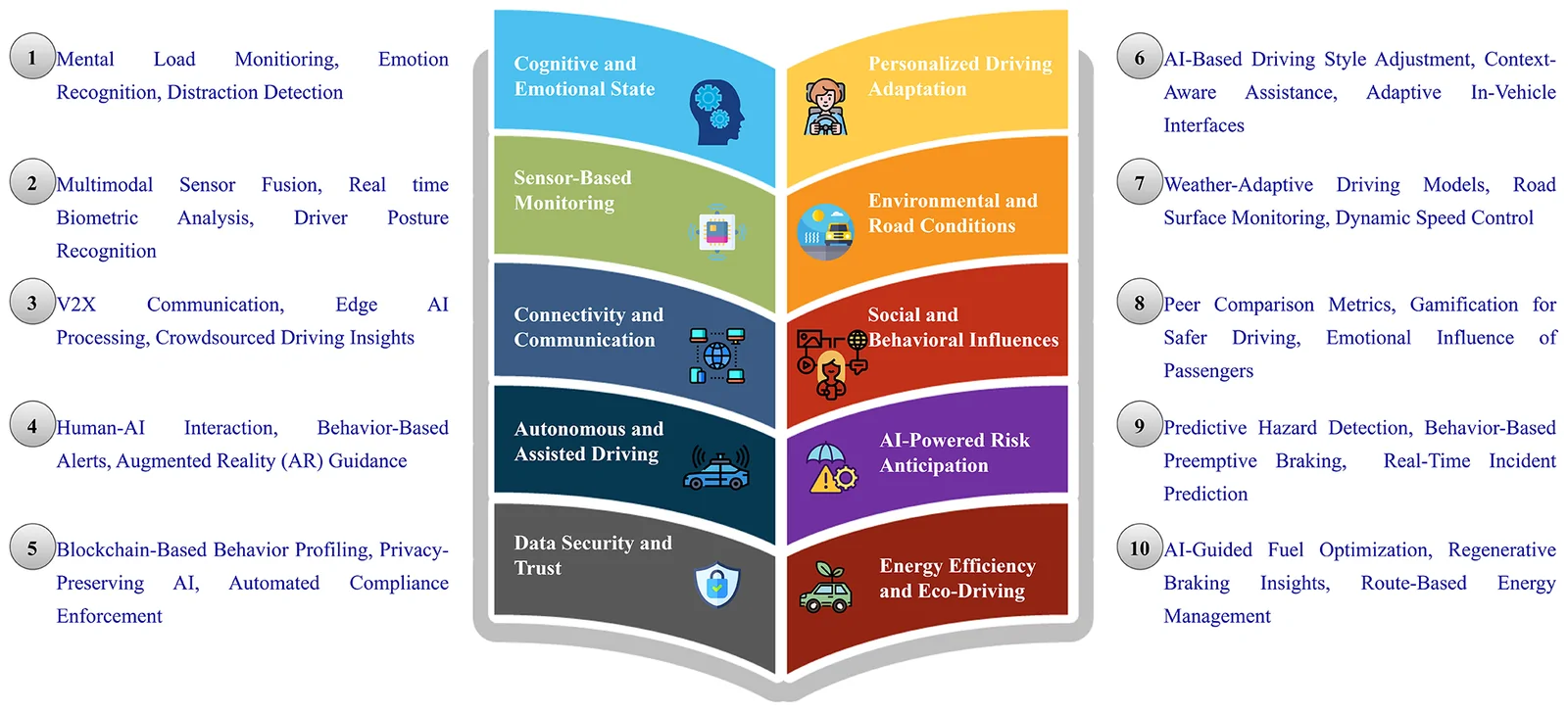
Factors influencing driver behavior.

#### 3.3.1 Comparative analysis and trade-offs of key technologies

Although machine learning, sensor fusion, biometric monitoring, and federated learning are all numerous technologies applied in driver behavior analysis, they differ greatly in terms of roles, maturity levels, and trade-offs.

The comparison in Table 7 is based on the outcomes from the literature, actual usage, and the claimed performance of each technology. All technologies are assessed with respect to accuracy, real-time capability, computational complexity, scalability, data privacy, and level of maturity. The capability of identifying or predicting driver behavior accurately and with low latency. Computational complexity is the amount of computing and memory that the technology needs. Scalability is the capacity to accommodate a growing number of drivers and vehicles, as well as data sources and behavioral tasks. Data privacy refers to the ability to secure sensitive driver and vehicle information in both data processing and sharing. Maturity level indicates the level of development and practical use, from experimental use to deployment. For these criteria, the qualitative categories “Very High,” “High,” “Medium,” and “Low” are used to show the relative capability of each technology with respect to the criterion. Medium to High” and “Low to Medium” are intermediate categories that represent the values between the respective levels.

**Table 7:** Comparative overview of key technologies in driver behavior analysis.

Technology	Accuracy	Real-time capability	Computational complexity	Scalability	Data privacy	Maturity level	Limitations
Machine learning (ML) (Uddin et al., 2025)	High	Medium to High	Medium	High	Medium	Mature	Requires large labeled datasets; vulnerable to overfitting
Deep learning (DL) (Gupta et al., 2024)	very high	Medium	High	Medium	Medium	Emerging in vehicles	High computational cost; lacks interpretability
Sensor fusion (Liu et al., 2020)	High	High	High	Medium	Neutral	Deployed in ADAS	Expensive hardware; calibration and redundancy issues
Biometric monitoring (Zhou S. et al., 2024)	Medium	Medium	Medium	Low	Low	Experimental	Intrusive; privacy-sensitive; sensor drift and variability
Federated learning (FL) (Tabatabaie and He, 2024)	Medium	Medium	Medium to high	High	Very high	Early-stage in vehicles	Limited by device heterogeneity and communication constraints
Edge computing (Wang et al., 2023)	Medium	Very high	Low to medium	Medium	High	Growing adoption	Limited onboard resources; needs optimization

The standard machine learning algorithms typically operate at an accuracy range of between 85 and 95%, and this is considered to be High. Conversely, the biometric and federated learning models tend to be subject to inter-user and inter-system variability, which leads to a Medium level of accuracy because of personalization and data quality constraints. The real-time capability is evaluated in terms of responsiveness of the system to dynamic driving conditions. Edge computing and sensor fusion usually offer sub-second response time and have already found applications in commercial Advanced Driver Assistance Systems (ADAS), which accords it a score of Very High. In the meantime, deep learning and biometric monitoring systems need more processing and can be delayed, which can only be rated Medium. Computational complexity is a measure of processing resources and memory required to do inference; deep learning and multi-sensor fusion are resource-intensive (high), traditional machine learning models are minimally resource-intensive (Medium), and optimized edge inference systems (low) are resource-intensive. Scalability: scalability is how easy it is to deploy to a variety of vehicles or platforms. Federated learning can be used with large-scale decentralized learning (Very High), and traditional ML algorithms can be deployed with ease in production (High), whereas biometric systems are frequently limited by personalization requirements, limiting scalability to (Medium). Another important metric is data privacy, where federated learning performs better because it has a local training paradigm (“Very High”), as edge computing helps to improve privacy through the reduction of data transmission (High), and centralized methods based on biometric data are more dangerous and lead to a lower category. Lastly, the maturity level is classified based on industry readiness. Machine learning and sensor fusion are mature and commercially deployed (Mature), deep learning and federated learning are actively emerging but not yet deployed in cars (Emerging), biometric and physiological monitoring systems are still largely experimental and have not yet been widely integrated into consumer automotive platforms.

### 3.4 Datasets

The datasets of driver behavior are vital in conceptualizing, examining, and enhancing road safety, autonomous driving technology, and human-computer interface in automotive. These datasets are able to capture different facets of driving behavior such as naturalistic driving patterns, distracted driving, traffic patterns, and situations that are prone to accidents. They usually contain multimodal sensor information like GPS, accelerator, gyroscope, LiDAR, and in-car camera footage to allow sophisticated behavioral forecasting and risk evaluation. Researchers use these datasets to build models for driver profiling, attention monitoring, accident prevention, and adaptive driver assistance systems. Table 8 lists key driver behavior datasets, their descriptions, and their usage.

**Table 8:** Datasets for analysis.

Category	Dataset name	Description	Applications	URL
Driver behavior and related datasets	Berkeley DeepDrive (BDD100K) (Yu et al., 2020)	A heterogeneous set of driving data consisting of 100K videos of different weather conditions, lighting, and driving situations.	Object detection, lane detection, behavior analysis, and multi-task learning in autonomous driving (Miraliev et al., 2023).	https://www.kaggle.com/datasets/solesensei/solesensei_bdd100k
	Oxford RobotCar dataset (Maddern et al., 2017)	A dataset that was gathered within 1 year, containing more than 1,000 km of urban driving data along with images, LiDAR, and GPS data.	Localization, mapping, perception, and trajectory prediction (Singh et al., 2022).	https://robotcar-dataset.robots.ox.ac.uk/
	NuScenes (Caesar et al., 2020)	A multi-modal dataset with 3D LiDAR point clouds, camera images, radar, and object annotations.	3D object detection, tracking, sensor fusion, and trajectory forecasting (Fong et al., 2022).	https://www.nuscenes.org/
	Argoverse (Chang et al., 2019)	A dataset with sensor data and high-definition maps for self-driving cars, covering urban driving scenes.	Motion forecasting, object detection, and HD map-based navigation (Wilson et al., 2023).	https://www.argoverse.org/
	Ford Multi-AV dataset (Agarwal et al., 2020)	A multi-agent autonomous vehicle dataset collected in diverse weather conditions, including LiDAR, GPS, and camera data.	Vehicle perception, localization, and seasonal adaptation for self-driving (Zhou W. et al., 2024).	https://avdata.ford.com/
	INTERACTION dataset (Zhan et al., 2019)	A dataset capturing human driver behavior, including cooperative and adversarial driving scenarios.	Behavioral prediction, motion planning, and traffic interaction modeling (Huang et al., 2026).	https://github.com/interaction-dataset/interaction-dataset
	UAH-driveset (Romera et al., 2016)	A dataset for analyzing driving behavior, including smartphone sensor data (GPS, accelerometer, gyroscope).	Driver behavior classification, distracted driving detection, and activity recognition (Liu W. et al., 2024).	https://github.com/Eromera/uah_driveset_reader
	Distracted driver detection dataset (state farm) (Anna et al., 2016)	A dataset containing images of drivers in various distracted states (texting, eating, adjusting radio, etc.).	Distracted driver detection, safety applications, and vision-based classification (Wang and Yao, 2024).	https://www.kaggle.com/competitions/state-farm-distracted-driver-detection/data
	NHTSA naturalistic driving study (SHRP2) (Russell et al., 2018)	A large-scale naturalistic driving dataset with real-world driving behaviors, accidents, and near-misses.	Traffic safety analysis, driver risk assessment, and accident prevention studies (Ali et al., 2023).	https://highways.dot.gov/safety/data-analysis-tools/rsdp/rsdp-tools/strategic-highway-research-program-shrp2
	Drive&Act dataset (Martin et al., 2019)	A multi-modal dataset for fine-grained driver behavior recognition using RGB, depth, and inertial sensors.	Driver action recognition, attention analysis, and in-vehicle activity monitoring (Shariff et al., 2023).	https://driveandact.com/
	Brain4Cars dataset (Jain et al., 2016)	A dataset that uses sensory fusion and deep learning for predicting driver intentions before actions occur.	Early action prediction, accident prevention, and human-machine interaction (Guo et al., 2023).	https://github.com/asheshjain399/ICCV2015_Brain4Cars
	Driving behavior dataset (DBD) (Yuksel et al., 2020)	A dataset containing various driving behavior data, including speed, acceleration, and road conditions.	Driver profiling, behavior prediction, and risk assessment (Hu et al., 2020).	https://data.mendeley.com/datasets/jj3tw8kj6h/2
Autonomous-driving perception datasets	Waymo open dataset (Sun et al., 2020)	A massive dataset of sensor data of autonomous vehicles, LiDAR, camera imagery, and HD maps.	Object detection, tracking, scene segmentation, motion prediction for self-driving cars (Hu et al., 2023).	https://waymo.com/open/
	LISA traffic light dataset (Jensen et al., 2016)	A dataset focused on detecting and recognizing traffic lights under various environmental conditions.	Traffic light detection, classification, and signal state prediction (Philipsen et al., 2015).	https://www.kaggle.com/datasets/mbornoe/lisa-traffic-light-dataset
	ApolloScape (Huang et al., 2018)	A large-scale dataset for autonomous driving with pixel-wise segmentation, lane markings, and depth information.	Semantic segmentation, road scene understanding, and lane detection (Shaikh and Faraz, 2026).	https://apolloscape.auto/
	Cityscapes dataset (Cordts et al., 2016)	A dataset focused on semantic urban scene understanding with pixel-wise labeled images.	Semantic segmentation, object detection, and urban scene analysis (Suryanto et al., 2025).	https://www.cityscapes-dataset.com/

## 4 Enabling technologies in driver behavior analysis

The mathematical expressions described in this section are typical formulations and are meant to convey the principle of the technologies and computational approaches described. They are not full mathematical algorithms or models for all algorithms or models in each category. As there are several models that can be used for the analysis of driver behavior, the formulations are shown selectively to give conceptual and mathematical background for the various approaches. The major enabling technologies for driver behavior analysis are shown in Figure 3, which include machine learning, deep learning, multimodal sensing, physiological-signal analysis, computer-based perception, and intelligent driver assistance technologies (Du et al., 2024; Engström et al., 2024). These technologies enable the derivation of behavioral information from vehicle, driver, physiological, visual, and environmental data. Recent works showcase the application of machine learning to multimodal physiological signals, modeling driver adaptation behavior and intelligent multimodal perception for driver-state and driver-behavior analysis (Tang et al., 2024; Giorgi et al., 2025).

**Figure 3:**
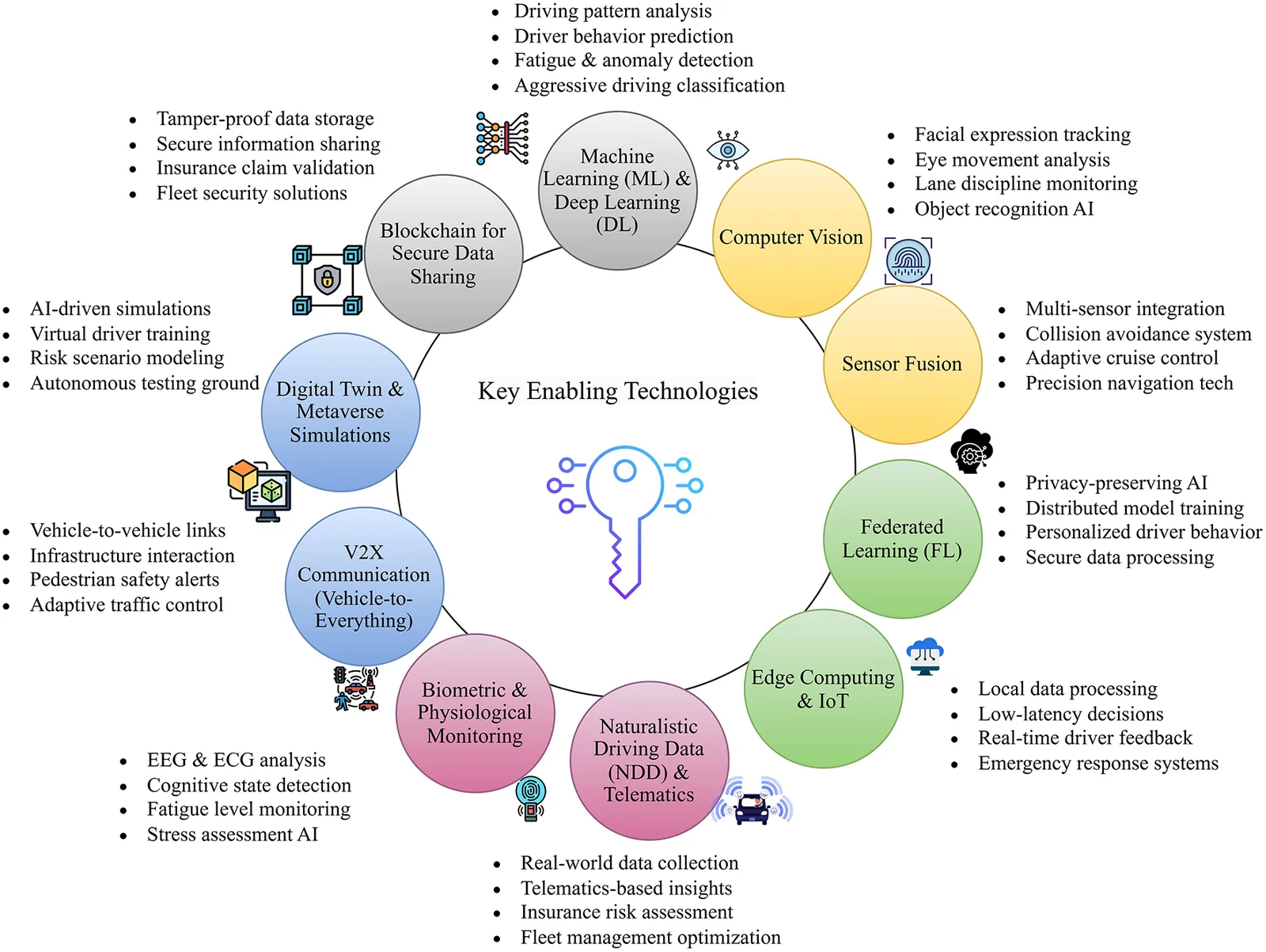
Enabling technologies for driver behavior analysis.

### 4.1 Machine learning (ML) and deep learning (DL)

ML and DL are widely used in driver behavior analysis to identify patterns, predict driver states, and provide real-time feedback. The use of ML algorithms on historical data can be used to classify or predict behaviors like distraction and drowsiness. DL uses deep neural networks for example convolutional neural networks (CNNs) to identify images, and long-short term memory (LSTMs) to process time-series data, to learn complex trends in driver behavior (Mengistu et al., 2025). Input data can include vehicle data, like GPS and speed, and driver data, like facial expressions. The idea is to use historical data to train models to classify or predict a driver's behavior. The product gives outputs of dynamic classifications or feeds, including drowsy/alert and aggressive/normal driving. Linear regression is a typical machine learning method based on regression and can be formulated as follows:


$$\begin{array}{l} y=w_{0}+w_{1}x_{1}+w_{2}x_{2}+\cdots +w_{n}x_{n} \end{array}$$ (1)

As shown in Equation (1), *y* denotes the prediction, *x*_1_, *x*_2_, …., *x*_*n*_ are the features. Neural networks are trained in DL to reduce a cross-entropy loss. As an illustration, a drowsiness detection system could be designed based on facial expressions and a CNN, with the help of which the driver would be informed about their drowsiness, whereas an aggressive driving detection model could be created to predict flirting with dangerous driving behaviors and provide the driver with instant feedback to avoid an accident.

### 4.2 Computer vision

Computer Vision involves a technology of reading and analyzing visual information from cameras or sensors that is instrumental in tracking the behavior of drivers. It assists in identifying the presence of distraction, fatigue, or risky behavior through analyzing facial expressions, head movements, and the surroundings of the vehicle. The input comprises images or video feeds from cameras installed inside and outside the vehicle. The role consists of image processing algorithms, such as facial recognition, object detection, and motion tracking, to determine driving conditions or possible dangers (Sun et al., 2025). The product provides real-time alerts or feedback, which may identify a drowsy driver or warn about obstacles on the road. Computer vision can be based on the working principle of extraction of features by such techniques as Convolutional Neural Networks (CNNs). Given the example below, the facial recognition formula for detecting eye closure (as a sign of drowsiness) can be written as:


$$\begin{array}{c} EAR=\frac{∥p_{2}-p_{6}∥+∥p_{3}-p_{5}∥}{2∥p_{1}-p_{4}∥} \end{array}$$ (2)

As shown in Equation (2), *p*_1_, *p*_2_, *p*_3_, *p*_4_, *p*_5_, and *p*_6_ represent the six facial landmarks around the eye, ∥*p*_*i*_−*p*_*j*_∥ is the Euclidean distance between two of the landmarks, and EAR is the eye aspect ratio. The lower the EAR value, the more closed the eyes are, which can be used to determine driver drowsiness. Facial analysis using CNNs for recognition of driver drowsiness and pedestrian recognition.

### 4.3 Sensor fusion

Sensor Fusion involves the merging of data from various sensors to enhance driver behavior analysis, including cameras, radars, and GPS. It helps identify distractions, fatigue, and risky behaviors in real time.

The input will have data from different sensors (e.g., video, radar, speed sensors) (Aravinth et al., 2025; Yaswanth et al., 2023). The role combines sensor data to isolate information and improve accuracy. The output makes good predictions of driver behavior. The Kalman filter is represented through four steps, given by Equations 3a–d, which describe state prediction, error covariance prediction, Kalman gain calculation, and state update, respectively.


$$\begin{array}{l} {\hat{x}}_{k|k-1}=A{\hat{x}}_{k-1|k-1}+Bu_{k} \end{array}$$ (3a)

$$\begin{array}{l} P_{k|k-1}=AP_{k-1|k-1}A^{T}+Q \end{array}$$ (3b)

$$\begin{array}{l} K_{k}=P_{k|k-1}H^{T}{\left(HP_{k|k-1}H^{T}+R\right)}^{-1} \end{array}$$ (3c)

$$\begin{array}{l} {\hat{x}}_{k|k}={\hat{x}}_{k|k-1}+K_{k}\left(z_{k}-H{\hat{x}}_{k|k-1}\right) \end{array}$$ (3d)

As shown in Equations (3a–d), the predicted state estimate at time k, ${\hat{x}}_{k|k-1}$ is the updated state estimate from the previous time step ${\hat{x}}_{k-1|k-1}$, A is the state-transition matrix, B is the control-input matrix, *u*_*k*_ is the control input, *P*_*k*∣*k*−1_ is the predicted error covariance matrix, Q is the process-noise covariance matrix, *K*_*k*_ is the Kalman gain, H is the measurement matrix, R is the measurement-noise covariance matrix, and *z*_*k*_ is the measurement received at time k. The new state estimate, ${\hat{x}}_{k|k}$ is the predicted state with the available sensor measurement.

Drowsiness detection is sensor-fused with facial and telemetry data, and collision avoidance is sensor-fused with radar, lidar, and camera data to detect obstacles.

### 4.4 Federated learning (FL)

Federated Learning (FL) allows training machine learning models on decentralized devices (i.e., in-vehicle systems) without exchanging raw data. This strategy provides greater privacy by storing sensitive information about the driver on devices locally, but also supports collaborative learning between vehicles (Eid Kishawy et al., 2024). It uses local data from in-vehicle sensors, i.e., steering angles, speed, and driver behavior data. The role trains a worldwide model through the aggregation of locally trained models, such that data does not leave the vehicle. The result is a common international model capable of foretelling or categorizing driver behavior. The conceptualization is that the working principle is that every vehicle updates its local model, and every so often, combines these updates to enhance the global model, by way of an aggregation algorithm such as Federated Averaging. The update step is:


$$\begin{array}{l} \begin{array}{c} w_{t+1}=\sum_{i=1}^{K}\frac{n_{i}}{N}w_{i}^{\left(t\right)} \end{array} \end{array}$$ (4)

As shown in Equation (4), *w*_*t*+1_ is the updated global model at communication round *t*+1, K is the number of participating clients, $w_{i}^{\left(t\right)}$ is the locally trained model of client iat round *t*, *n*_*i*_ is the number of training samples available at client i, and $N=\overset{K}{\underset{i=1}{\sum }}n_{i}$ is the total number of training samples across all participating clients. Thus, the weights of the local models can be proportional to the number of training samples for each local model.

One use case is personalized driver assistance systems, in which the global model will be adjusted to the driving styles of a specific individual in a variety of vehicles, and predictive maintenance, in which FL is used to predict the behavior of a vehicle without disclosing personal information.

### 4.5 Edge computing and IoT

The use of Edge Computing and IoT can allow a driver to analyze driver behavior and process data in real-time, with calculations occurring on the hardware of the vehicle, resulting in minimal latency. The input is the data from the IoT sensors (e.g., accelerators, GPS, cameras) (Lin et al., 2025). This data is processed and analyzed on the local level to give instant feedback. The output is in-time alerts or forecasts, i.e., unsafe driving behavior or fatigue.


$$\begin{array}{l} Anomaly\;Score=\;\frac{\left|x-\mu \right|}{\sigma } \end{array}$$ (5)

As shown in Equation (5), x represents the current data point, μ represents the average of the normal data, and σ represents the standard deviation. When the score goes above a predetermined limit, an anomaly is reported.

One of the applications of edge computing is in real-time driver safety, whereby edge computing processes vehicle and driver data to identify dangerous behaviors, including a sharp turn or excessive braking. The other use case is predictive maintenance, with IoT sensors monitoring any early signs of wear and tear in a vehicle or its potential failure; then proactive maintenance is performed.

### 4.6 Naturalistic driving data (NDD) and telematics

The term Naturalistic Driving Data (NDD) refers to natural driving data that is gathered in a real-life scenario on vehicles to give insight into driver behavior under normal conditions. Telematics is used to mean the combination of telecommunication and vehicle transportation information to track, control, and interpret driving habits on the fly (Xiang et al., 2024). The input includes NDD, which is gathered on vehicles (i.e., speed, acceleration, braking patterns, etc.), and telematics information that is gathered by GPS, sensors, and in-vehicle monitoring systems. This information is processed and measured by the function to determine different factors of driver behavior, including risk-taking or drowsiness. The output yields information on the performance of the drivers with risk assessment, driving pattern, and improvement suggestions. The principle of operation is based on data consolidation using different sensors and predicting or categorizing behavior using statistical models or machine learning algorithms. One of the formulas applied in telematics data analysis is:


$$\begin{array}{l} Risk\;Score=\;\frac{\sum_{i=1}^{n}Severity_{i}\times Frequency_{i}}{n} \end{array}$$ (6)

As shown in Equation (6), Severity_i_ is a measure of the risk of a given event happening (e.g., hard braking) and Frequencyi is the frequency of that particular event, and n is the number of events in total (Rizwan et al., 2016). One use case is driver risk profiling, wherein the NDD and telematics data are used to profile the drivers according to the risk factors, and fuel efficiency optimization, wherein driving habits are tracked to propose more efficient driving habits.

### 4.7 Biometric and physiological monitoring

Biometric and Physiological Monitoring implies monitoring and measuring the physiological indicators of drivers (heart rate, eye movement, and skin conductance) to determine their alertness, stress, and emotional condition. Such information aids in comprehending the physical and psychological state of the driver, which gives information on such behaviors as fatigue, distraction, or even aggression (Bisogni et al., 2024). The input is the data provided by biometric sensors (e.g., heart rate sensors, eye tracking cameras, skin conductance sensors). The functionality interprets these biological indicators in order to determine the indicators of exhaustion or stress and evaluate the general condition of a driver. The output may contain real-time notifications or reports on the actual condition of the driver, including drowsiness detectors or stress detectors. The principle of operation may include algorithms of time-series analysis of physiological data to identify anomalies. One of the formulas applied in the analysis of heart rate variability (HRV) is:


$$RMSSD=\sqrt{\frac{1}{N-1}{\sum_{i=1}^{N-1}\left(\;RR_{i+1}-RR_{i}\;\right)}^{2}\;}$$ (7)

As shown in Equation (7), *RMSSD* is the root mean square of the successive differences between successive heartbeat intervals, *RR*_*i*_ is the i-th successive RR interval in seconds or milliseconds, *RR*_*i*+1_ is the successive RR interval, and N is the number of RR intervals being considered. RMSSD is a time-domain measure of heart rate variability, and it can be used to describe changes in autonomic cardiac activity.

Applications are in drowsiness detection, where biometric data such as eye-tracking and heart rate can be used to determine driver fatigue, and stress monitoring, where changes in skin conductance and heart rate can be used to determine high levels of stress whilst driving.

### 4.8 V2X communication (vehicle-to-everything) in driver behavior analysis

V2X communication allows cars to communicate with each other, with infrastructure, pedestrians, and networks in order to improve safety and driving optimization. The input will include information about the neighboring cars, infrastructure, and pedestrians. This information is processed by the function to enhance decision-making and to warn drivers (Chen et al., 2024). The output contains real-time feedback, e.g., collision warnings or route alteration. Its working principle implies the exchange of data in such protocols as DSRC or C-V2X. For collision risk:


$$\begin{array}{l} \begin{array}{c} TTC=\frac{d}{v_{1}-v_{2}},\;v_{1}>v_{2} \end{array} \end{array}$$ (8)

As shown in Equation (8), TTC denotes the estimated time to collision, and d is the longitudinal distance between the two vehicles, *v*_1_ the velocity of the following vehicle and *v*_2_ the velocity of the vehicle in front. The condition *v*_1_>*v*_2_ means that the approaching vehicle is in front of the vehicle under study. The lower the TTC, the less time available to react to a possible collision.

Examples of use cases are collision avoidance, where V2X traffic can avoid accidents due to exchanging real-time traffic and hazard data, and traffic flow optimization, where V2X traffic will be used to adjust the driving speed and route to alleviate congestion.

### 4.9 Digital twin and Metaverse simulations in driver behavior analysis

Digital Twin technology is a technology that builds virtual models of a car and its surroundings to act as a simulation of the real world and forecast the behavior of drivers. Metaverse simulations further apply this idea to simulated environments that are immersive and offer an experience of testing and analyzing driver behavior under different conditions, without any risk of harm to the real world (Ma et al., 2024). Its input is real-time data of vehicles and the surroundings, including driving habits, traffic, and climate. The role develops a virtual simulation of the car and the environment to simulate interactions and make predictions. The output contains computer simulations of driving behavior, safety situations, and performance measures. The principle of operation is based on sensor data, as it will update the digital twin in real time, and the metaverse will involve the integration of these models into a simulated environment. A state-space model for predicting vehicle dynamics could be used for simulation:


$$\begin{array}{l} ẋ=Ax+Bu \end{array}$$ (9)

As shown in Equation (9), ẋ is the time derivation of the state vector, where *x* is the state of the vehicle (e.g., position, speed); *u* is the control (e.g., acceleration); *A* and *B* are matrices that give the dynamics of the vehicle. They are used as a training environment in driver training with immersive simulations and in predictive maintenance, where virtual models can predict the possibility of a vehicle having issues based on the simulated driving conditions.

### 4.10 Blockchain for secure data sharing in driver behavior analysis

Blockchain technology offers a decentralized and immutable registry to safely share the driver behavior data with vehicles, infrastructure, and other stakeholders. It provides data privacy, integrity, and transparency, making it well-suited to handle sensitive driver information and facilitate safe data exchange. The input consists of data from vehicles, which includes driving patterns, location, and sensor data (Zhu et al., 2025). The role captures and certifies transactions within a decentralized and encrypted register, and data cannot be altered. The result will be a secure and auditable history of driver behavior which can be provided to the authorized parties. The principle of operation consists of the development of a block of transaction data, combined with links to the prior ones, making them immutable. An example of the hash-linking mechanism used to maintain blockchain data integrity is:


$$\begin{array}{l} \begin{array}{c} H_{i}=SHA256\left(H_{i-1}∥D_{i}\right) \end{array} \end{array}$$ (10)

As shown in Equation (10), *H*_*i*_ is the hash value of the current block; *H*_*i*−1_ is the hash value of the previous block; *D*_*i*_ is the data present in the current block; SHA256 is the cryptographic hash function, - and || is the concatenation of the hash of the previous block and the data of the current block. This hash linking system helps keep blockchain records intact and tamper-evident. Examples of use cases are secure data exchange among vehicles in V2X communication and driver performance monitoring, in which blockchain will ensure that data is tamper-free and transparent.

Table 9 summarizes the symbols, variables, parameters, abbreviations, and measurement units used in the mathematical formulations. Physical quantities are assigned their corresponding measurement units, while mathematical quantities are identified by their appropriate type. Some symbols are used in different equations with context-specific meanings. For example, *x* represents the current data point in the anomaly-score equation, whereas *x* represents the vehicle state vector in the state-space model in Equation 9 Similarly, the *N* denotes the total number of training samples in the FedAvg formulation and the number of RR intervals considered in the.

**Table 9:** Nomenclature of symbols, variables, parameters, abbreviations, and measurement units.

Symbol/abbreviation	Definition	Measurement unit/type
*Y*	Predicted output of the linear regression model	Output-dependent
*w*_0_	Intercept of the linear regression model	Model parameter
*w*_*i*_	Weight associated with input feature *x*_*i*_	Model parameter
*x*_*i*_	*i*-th input feature	Feature-dependent
*EAR*	Eye aspect ratio	Dimensionless
*p*_1_, …, *p*_6_	Six eye landmark points used for EAR calculation	Pixel coordinates
∥*p*_*i*_−*p*_*j*_∥	Euclidean distance between two eye landmarks	Pixels
${\hat{x}}_{k|k-1}$	Predicted state estimate at time *k*, based on measurements available up to time *k*−1	State-dependent
*A*	State-transition matrix	Matrix
*B*	Control-input matrix	Matrix
*u*_*k*_	Control input at time *k*	Input-dependent
*P*_*k*∣*k*−1_	Predicted error covariance matrix at time *k*, based on measurements up to time *k*−1	Matrix
*Q*	Process-noise covariance matrix	Matrix
*K*_*k*_	Kalman gain at time *k*	Matrix
*H*	Measurement matrix	Matrix
*R*	Measurement-noise covariance matrix	Matrix
*z*_*k*_	Measurement at time *k*	Measurement-dependent
*w*_*t*+1_	Updated global model parameters at communication round *t*+1	Model parameter
$w_{i}^{\left(t\right)}$	Local model parameters of the client *i* at round *t*	Model parameter
*K*	Number of participating clients	Count
*n*_*i*_	Number of training samples at client *i*	Count
*N*	Total number of training samples	Count
*x*	Current data point used in anomaly-score calculation	Data-dependent
μ	Mean of the normal data	Data-dependent
σ	Standard deviation of the normal data	Data-dependent
*Severity*_*i*_	Severity associated with risk event *i*	Dimensionless
*Frequency*_*i*_	Frequency of risk event *i*	Occurrences
*i*	Index of a risk event or observation	Index
*RMSSD*	Root Mean Square of Successive Differences	Milliseconds
*RR*_*i*_	*i*-th successive RR interval	Milliseconds
*RR*_*i*+1_	Subsequent RR interval	Milliseconds
*TTC*	Time to collision	seconds
*d*	Longitudinal distance between two vehicles	meters
*v*_1_	Velocity of the following vehicle	meters per second
*v*_2_	Velocity of the vehicle ahead	meters per second
ẋ	Time derivative of the vehicle state vector	State-dependent/s
*x*	Vehicle state vector in the state-space model	State-dependent
*u*	Vehicle control input	Input-dependent
*H*_*i*_	Hash value of the current blockchain block	256-bit hash
*H*_*i*−1_	Hash value of the previous blockchain block	256-bit hash
*D*_*i*_	Data contained in the current blockchain block	Data-dependent
*SHA*256	Secure Hash Algorithm with 256-bit output	Cryptographic algorithm
∥	Concatenation operator	Mathematical operator
*ML*	Machine learning	Abbreviation
*DL*	Deep learning	Abbreviation
*FL*	Federated learning	Abbreviation
*NDD*	Naturalistic driving data	Abbreviation
*IoT*	Internet of things	Abbreviation
*V*2*X*	Vehicle-to-Everything	Abbreviation
*HRV*	Heart-rate variability	Abbreviation
*CNN*	Convolutional neural network	Abbreviation
*DRL*	Deep reinforcement learning	Abbreviation
*EMS*	Energy management strategy	Abbreviation
*FCHEV*	Fuel cell hybrid electric vehicle	Abbreviation

RMSSD formulation. These meanings differ by equation.

## 5 Applications of driver behavior analysis in social systems

The key applications of driver behavior analysis are shown in Figure 4: road safety and risk assessment, driver identification and profiling, fuel and energy efficiency, personalized driving assistance, usage-based insurance, and intelligent transportation systems. Data from vehicle sensors, telematics, physiology, and visual data can be used to detect driving styles, to evaluate driver risk, to enhance fuel efficiency, and to give individual feedback. Recent research shows how to use data and machine learning techniques for driver profiling, safety assessment, prediction of vehicle fuel consumption, adaptive assistance systems, and intelligent vehicle control (Mobini Seraji et al., 2025; Qu et al., 2024; Maktoubian et al., 2024).

**Figure 4:**
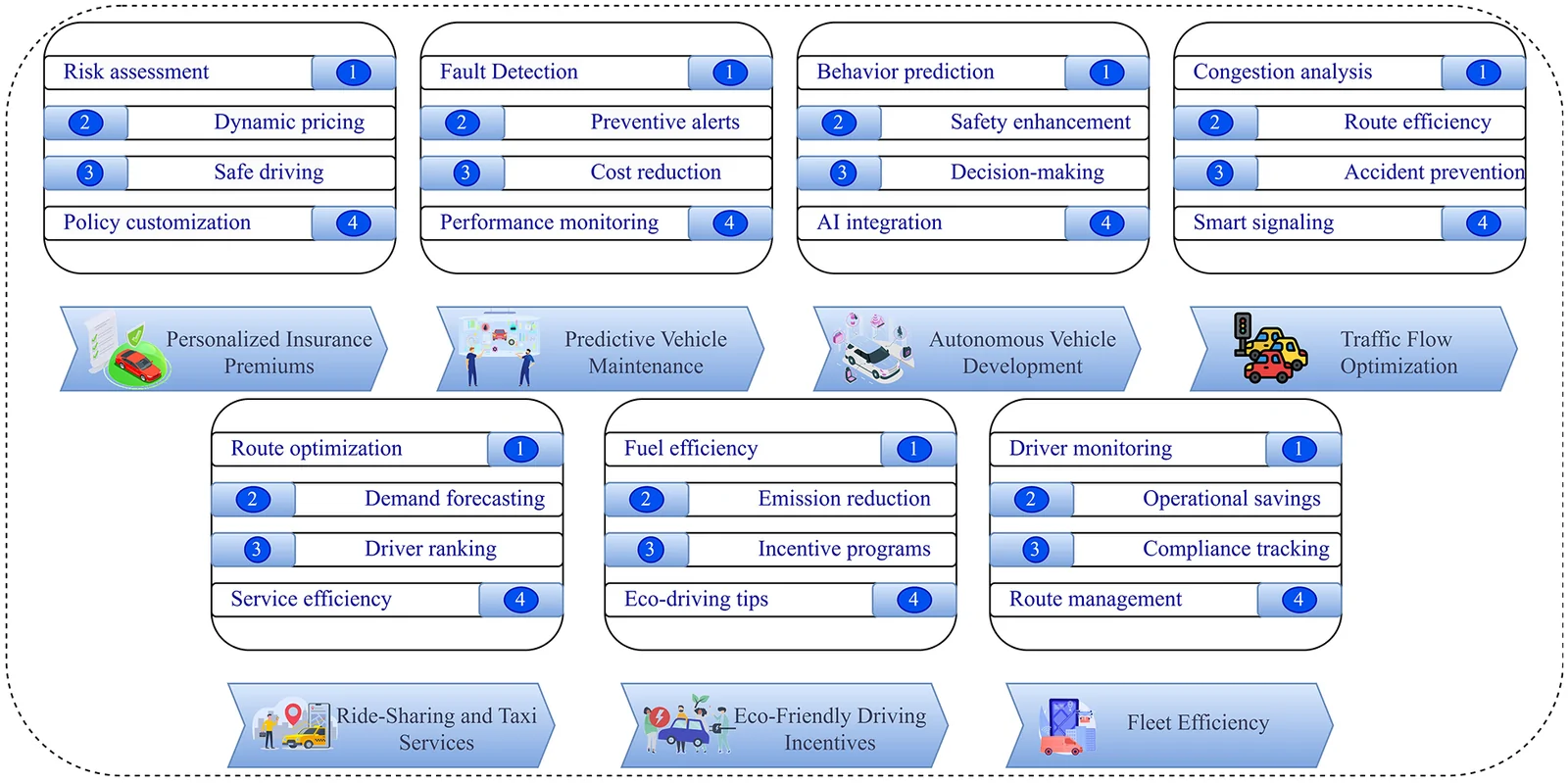
Driver behavior analysis applications.

### 5.1 Personalized insurance premiums

Personalized Insurance Premiums are those types of insurance in which an individual is charged on the basis of driving behavior as opposed to a set risk category (Yin and Chen, 2018). This will help ensure fair pricing, as safe drivers are rewarded, and premiums are set higher for those who have risky behavior. Conventional risk models of insurance involve generalization of risk factors, which in most cases result in unfair pricing of premiums (Masello et al., 2025). Usage-Based Insurance (UBI) is possible through driver behavior analysis, as it gauges the driving patterns in real-time, including speed, braking, acceleration, and the level of distraction during critical moments. This assists insurance companies in offering reasonable and competitive premiums, in which safe drivers benefit with reduced premiums and those who act in a risky manner face a penalty.

Case study 1: snapshot program (USA) provided by Progressive (USA) is a telematics program that monitors driving behavior and reviews premiums on the basis of safe driving behavior. Violations like low speed and smooth acceleration and braking are given discounts to drivers.

Case study 2: the smart telematics (Europe) proposes a mile-based insurance by Allianz, which saves money on low mileage and safe drivers. The system monitors driving behavior, and the premiums that are given to the individual are reduced for those individuals who adopt a consistent and responsible driving behavior.

### 5.2 Predictive vehicle maintenance

Predictive Vehicle Maintenance is based on real-time data on driver behavior to identify possible mechanical problems and prevent breakdowns. Predictive maintenance can be used to optimize the schedule of services by examining trends in aggressive acceleration, harsh braking, and excessive idle time and minimizing the cost of repairing the car, as well as avoiding the occurrence of failures. Violent driving behavior such as sudden braking, aggressive accelerating, and over-idling can cause early wear and sudden vehicle breakdowns (Zhang et al., 2019; Yaswanth and Babu, 2025). The analysis of driver behavior contributes to the early identification of mechanical problems in order to provide the opportunity to carry out routine maintenance prior to the development of expensive failures. This enhances the reliability of the vehicles and increases their lifespan.

Case study 1: the vehicle health monitoring used by Uber will inform drivers about possible problems dependent on aggressive driving behavior and will prevent unforeseen breakdowns. The system suggests maintenance solutions, which guarantee the performance of the fleet and the safety of drivers.

Case study 2: DHL fleet telematics system relies on driver behavior data in the anticipation of brake and engine maintenance to minimize downtime. DHL ensures reduced repair costs and chances of extending vehicle life by tracking driving styles and mechanical stress.

### 5.3 Autonomous vehicle development

The development of Autonomous Vehicles is based on human driver behavior analysis to train AI models to drive autonomous vehicles. Learning human driving behavior can enable autonomous vehicles (AVs) to make viable and predictable decisions to enhance the process of lane changing, braking, and obstacle detection (Altun and Celenk, 2017). To blend seamlessly with human driver traffic, autonomous vehicles (AVs) should learn behavioral patterns of human drivers to predict human behavior and make better decisions (Ma et al., 2020). Real-life driver behavior data is used to train AI models that are used to improve lane changing, braking, and obstacle detection in AVs.

Case study 1: self-driving AI training at Waymo involves real-world driver data to educate AVs on how to react to road driving situations. The system keeps improving its algorithms through human interactions in various traffic situations.

Case study 2: Tesla Autopilot Learning System gathers and examines human driving behaviors to perfect autonomous driving choices. The system makes changes based on the different driving styles and promotes safety using driver feedback.

### 5.4 Traffic flow optimization

Traffic Flow Optimization uses the data on driver behavior to improve the operation of traffic signal, lessen the congestion, and also improve the efficiency of the road on the whole (Kamal et al., 2014). Through the study of real time driving behavior, the adaptive traffic systems can change the signals dynamically to enhance movement of the traffic. Traffic congestion increases time, fuel use, and emissions. The analysis of driver behavior assists in the performance of traffic lights, adjustment of speed limits, and creation of traffic jams dynamically through the examination of current driver behaviors.

Case study 1: Singapore Smart Traffic Light System is a system that employs AI-based control of driver behavior to decide how traffic lights respond to different conditions and minimize congestion. The system takes into consideration the peak hours and adjusts the time of traffic lights dynamically to facilitate better traffic flow.

Case study 2: Los Angeles Adaptive Traffic Control System (ATCS) is a traffic control technology that identifies driving patterns and adjusts traffic light timing in real-time to enhance the efficiency of traffic. The system monitors the amount of congestions on the road hence facilitating the efficient operation of traffic.

### 5.5 Ride-sharing and taxi services

Ride-Sharing and Taxi Services apply the driver behavior analysis that ensures passenger safety, fuel efficiency and enhanced service quality. Braking, acceleration, and monitoring and speed patterns assist in unsafe driving determination and high customer satisfaction ratings (Psaraftis et al., 2024). The safety of passengers and effective driving are very important to ride-sharing solutions. Analysis of driver behavior assists in detection of aggressive driving, fuel efficiency, and high customer satisfaction ratings.

Case study 1: Lyft has a system called the Driver Score System that tracks braking, acceleration, and speed to rank drivers and disable those that do not qualify as safe. This boosts passenger safety and promotes responsible driving.

Case study 2: Ola is an AI-powered ride monitoring system that employs driver analytics to ensure smooth rides and provides incentives to safe drivers. The system monitors driving behavior so that it can offer individual training and incentives.

### 5.6 Eco-friendly driving incentives

Eco-Friendly Driving Incentives promote fuel-efficient and environmentally friendly driving patterns by penalizing acceleration, speed, and idling hours. These incentives assist in cutting down on carbon emissions and minimizing the cost of fuel. Sustainable driving reduces fuel use and emissions (Aldhanhani et al., 2024). Behavior analysis assists in encouraging environmentally friendly driving behaviors like gentle acceleration, optimum speed, and low engine idling.

Case study 1: hybrid driving rewards is a program by Toyota that will encourage the owners of hybrid cars to maintain efficient driving habits to ensure they have a high level of mileage. Those drivers who embrace the concept of eco-driving are given tokens such as fuel discounts.

Case study 2: the green driving initiative in Germany is a tax incentive given to all drivers who are environmentally friendly, and their habits are tracked using telematics. The system evaluates driving efficiency and rewards low-emission driving behavior.

### 5.7 Fleet efficiency

Fleet Efficiency solutions involve the analysis of driver behavior to enhance fuel consumption and minimize maintenance expenses and deliver fuel efficiency (Pozueco et al., 2018). Observing severe braking, idling, and speed fluctuations improves performance and minimizes costs. Fleet managers use driver behavior analysis to optimize fuel consumption, minimize maintenance expenses, and shorten delivery times. Monitoring rough braking, idling, and changes in speed assists in enhancing efficiency in the fleets as well as lowering the operational costs.

Case study 1: FedEx Smart Fleet Monitoring applies AI in monitoring driver behavior and fuel consumption. The system assists the operators of the fleet to minimize unwarranted idling and enhance the efficiency of the routes.

Case study 2: Amazon Delivery Fleet Analytics uses the pattern of drivers to enhance their efficiency in routes and decrease vehicle wear. The system finds the best driving behavior, which reduces fuel expenses and enhances speed of delivery.

## 6 Research projects and use cases

Global-level projects contribute to the advancement of driver behavior analysis through collaborative research and the development of intelligent transportation technologies. The major contributions of ongoing global-level projects are summarized in Table 10.

**Table 10:** Contributions of global-level ongoing projects.

Project	AI/ML	Edge Computing	IoT/V2X	Federated Learning (FL)	Blockchain	Biometric Sensors	AR/VR Simulation	Digital Twin	BEHAVIOR MODELING	HMI	Telematics	Fatigue/Distraction Detection	Scenario-BasedEvaluation	Explainable AI (XAI)	Trust and Acceptance
AUTOPILOT (EU)	✓	✓	✓						✓	✓	✓		✓		✓
DRIVE C2X (EU)			✓							✓	✓		✓		✓
ADAPTIVE (EU)	✓								✓	✓					✓
VI-DAS (EU)	✓								✓	✓		✓			
U-DRIVE (EU)	✓		✓						✓		✓				
BERTHA (EU, 2023–26)	✓			✓			✓	✓	✓	✓			✓	✓	✓
iDriving (EU, 2024–26)	✓	✓	✓					✓	✓		✓		✓	✓	
Safe HTI (EU, 2025+)	✓								✓	✓		✓	✓	✓	✓
Scenario-Based CCAM (EU)	✓	✓	✓				✓	✓	✓	✓			✓	✓	✓
FITDRIVE (EU, till 2025)	✓	✓	✓			✓			✓		✓	✓			
SHRP2 NDS (USA)	✓								✓		✓		✓		
J-MaaS (Japan)	✓		✓					✓	✓	✓	✓			✓	✓
KoROAD (South Korea)	✓	✓	✓			✓			✓		✓	✓			
ADVI (Australia)	✓	✓						✓	✓	✓		✓	✓	✓	✓
iRASTE (India)	✓	✓	✓		✓			✓	✓		✓	✓		✓	

### 6.1 European Union (EU) projects

#### 6.1.1 Autopilot

The AUTOPILOT project aims to implement IoT and V2X communication to provide autonomous driving functions. It uses networked infrastructure and live data transmission to streamline mobility in the city and its coordination. Through cloud platforms and massive testbeds, AUTOPILOT confirms the promise of IoT-enhanced automation to enhance the safety and efficiency of traffic. The EU, through the Horizon2020 program, funded the project.

#### 6.1.2 Drive c2x

DRIVE C2X is dedicated to carrying out the validation of the cooperative systems of V2V and V2I communication to improve road safety. The project revealed the efficiency of hazard warning and intersection safety technologies based on large-scale field experiments in Europe. It had a modular architecture that facilitated cross-country interoperability testing of cooperative intelligent transport systems. It was funded through the EU FP7 program.

#### 6.1.3 Adaptive

The ADAPTIVE project aims at developing adaptive Human-Machine Interfaces (HMI) that react to driver workload and to the cognitive states. The system is dynamically customized to offer feedback and intervention strategies through the use of machine learning models. The aim is to enhance safety for humans in semi-autonomous cars by using behavior-sensitive interfaces. Horizon2020 supported the project.

#### 6.1.4 Vi-das

VI-DAS focuses on a refined vision driver aid, which uses computer vision with sensor fusion, and it is used to track drowsiness, distraction, and the environment. The system promotes situation awareness toward proactive safety intervention. VI-DAS is an investment funded by Horizon2020 and is involved in the creation of real-time driver behavior monitoring systems.

#### 6.1.5 U-drive

U-DRIVE is a naturalistic driving experiment whereby telematics and behavior data are utilized to evaluate the risk of driving in the real world. It uses statistical patterns on big, uncontrolled driving data, determining behavioral patterns associated with the cause of accidents. The EU-FP7-supported project is an information source for future adaptive safety system policies and design.

#### 6.1.6 Bertha

BERTHA focuses on human-like autonomous driving based on the development of Bayesian behavioral models. It combines human-in-the-loop experimentation and simulation models like CARLA, which allow replication of realistic behavior. The project helps to explain and assess scenarios and validate cross-domain. It is financed by Horizon Europe and operates up to 2026.

#### 6.1.7 iDriving

The iDriving project will improve intelligent transportation by modeling infrastructure-aware behavior. It applies AI, IoT sensors, and digital twin environments to contextualize vehicle decision-making. Horizon Europe supports the project, which focuses on data fusion to analyze risks through scenarios.

#### 6.1.8 Safe HTI

Safe HTI addresses safe handovers and manual-to-automated driving transitions. It discusses the interface design and interaction between humans and technology, real-time attention tracking, and adaptive interface design. The project will provide security and confidence when automating dynamically on different automation levels. It is among the future calls of Horizon Europe in 2025.

#### 6.1.9 Scenario-Based CCAM

The current project aims to test connected and automated mobility (CCAM) through setting up scenario-based testing. It integrates HMI, digital twins, and simulation devices to verify safety, trust, and behavioral reactions. It is concerned with developing transport systems that possess dynamic requirements of interaction. The Horizon Europe funds the project.

#### 6.1.10 FITDRIVE

FITDRIVE is a biometric driver monitoring (EEG, EMG) and AI app to identify fatigue and brainwork of a driver. It is focused on preventing accidents by offering individual feedback and behavioral risk evaluation. The project performs trials in various countries of the EU and incorporates adaptive safety interventions. It is a project funded by Horizon 2020 and will end in 2025.

### 6.2 Other projects and programs

#### 6.2.1 SHRP2 NDS

The SHRP2 Naturalistic Driving Study is a large project carried out in the United States, and it involves the collection of multi-modal driving data used to study behavioral factors that lead to road crashes. Having more than 3,500 drivers and 50 million miles of recorded data, the project uses statistical modeling to learn about actual driving behavior in the real world. It aids in the development of safety policies, insurance policies, and advanced driver assistance systems. The U.S. Federal Highway Administration leads the research.

#### 6.2.2 J-MaaS driver behavior program

J-MaaS is a program initiated in Japan, aimed at integrating driver behavior modeling into Mobility-as-a-Service (MaaS) ecosystems. It applies AI and multimodal transport analytics to anticipate user intent, maximize ride-sharing networks, and increase comfort among passengers. The Ministry of Land, Infrastructure, Transport and Tourism in Japan manages this project as part of the country's smart mobility transformation activities.

#### 6.2.3 KoROAD smart driver monitoring

The Korea Road Traffic Authority (KoROAD) is a South Korean national project to install AI-based driver monitoring systems. It incorporates biometric sensors to identify driver fatigue, driver distraction, and emotional stress in real-time driving conditions. The project will address the issue of improving road safety on highways and urban roads by actively focusing on the identification of risk-inducing behavioral patterns.

#### 6.2.4 ADVI driver monitoring trials

In Australia, the ADVI (Australian Driverless Vehicle Initiative) is a project carrying out driver behavior experiments to measure the behavior of attention, reaction, and handover in semi-autonomous driving. The project deals with human-in-the-loop testing in order to comprehend trust in automation and develop policy frameworks to integrate AVs. The results are added as national guidelines regarding automated driving technologies that should be deployed safely.

#### 6.2.5 iRASTE

iRASTE is a road safety project in India that uses an AI-based system, combining computer vision, sensor fusion, and behavioral modeling to identify driver risk in real time. It makes use of smart city infrastructure and telematics data to create early alerts and prescribe actions to take. It is the joint venture between NITI Aayog, IIIT-Hyderabad, Intel, and Government of Telangana as the aim of the project is to decrease the number of road accidents by implementing intelligent intervention systems.

## 7 Lessons learned, open issues, challenges

Driver behavior analysis is an important issue for developing intelligent mobility systems. The key findings, open issues, challenges, and future research directions from the reviewed literature are discussed here. It shows that the studies reviewed in this work can improve the accuracy and real-time responsiveness of driver behavior analysis systems through deep learning, edge computing, and sensor fusion. These technologies aid the detection of distracting, fatiguing, and aggressive behaviors, whilst minimizing processing delay and increasing the adaptability of the technology to varying driving scenarios. Even with these technological advancements, existing behavior monitoring systems for drivers are not integrated and scalable enough to monitor several driver behaviors at the same time. The challenges also include the generalization of models in varying driving situations, maintaining privacy in data processing, and minimizing biases in AI models. These limitations highlight the need for further research into decentralized architectures, effective training using heterogeneous datasets, and fairness-aware AI models. These key findings, open issues, challenges, and future research directions are summarized in Table 11.

**Table 11:** Open Issues, challenges, and future directions.

Open Issue	Description	Challenges	Future directions	Impact	Importance
Data privacy and security	Protecting sensitive driver data during collection, transmission, and storage.	Risk of data breaches, unauthorized access, and identity theft.	Use blockchain for secure data exchange and federated learning for decentralized model training.	Enhances trust and compliance in intelligent transport systems.	High – Critical for public acceptance, legal compliance, and ethical deployment.
Real-time processing	Analyzing vast sensor data with minimal latency.	High computational demand and dependency on cloud resources.	Use edge computing with low-latency hardware and 5G networks for faster in-vehicle processing.	Enables immediate decision-making for ADAS and safety alerts.	High – Essential for safety-critical applications and autonomous systems.
Sensor reliability	Inaccuracy in sensor data due to environmental interference.	Fog, glare, and rain can degrade sensor performance.	Employ sensor fusion with AI-based correction algorithms.	Improves robustness and reduces false alarms in behavior detection.	Medium – Needed for consistent performance in diverse driving conditions.
Generalization	Adapting AI models across diverse geographies and driving styles.	Poor model performance on unseen environments.	Use transfer learning and continuous self-adaptation mechanisms.	Boosts model scalability and accuracy in global applications.	High – Necessary for commercial deployment across countries and vehicle types.
Data standardization	Lack of uniformity in data formats across systems.	Difficulty in integrating datasets from different manufacturers.	Adopt open standards like Automotive Grade Linux (AGL) or Open Telematics Framework (OTF).	Facilitates collaboration and integration across platforms.	Medium – Enables ecosystem interoperability and cross-vendor innovation.
Scalability of FL	Managing federated learning across heterogeneous vehicles.	Varied network speeds and processing power in different cars.	Use asynchronous update strategies and compression for communication efficiency.	Expands secure learning to large-scale fleets.	Medium – Supports widespread adoption of privacy-aware analytics.
Bias in AI models	Models may unfairly penalize certain driver groups.	Dataset imbalance leads to discriminatory predictions.	Implement fairness-aware training and regular bias audits.	Ensures equitable treatment and model transparency.	High – Critical for ethical AI and regulatory compliance.
User acceptance	Resistance to monitoring and AI recommendations.	Privacy concerns and mistrust in system feedback.	Use gamification, personalized feedback, and explainable AI (XAI).	Increases user engagement and system effectiveness.	High – Key to successful adoption of AI-based behavior monitoring.
Regulatory compliance	Adhering to regional and international AI/data laws.	Complex, evolving legal frameworks for data usage and liability.	Develop compliance-aware frameworks and collaborate with regulators.	Prevents legal conflicts and ensures safe deployment.	High – Essential for long-term viability of intelligent mobility systems.

### 7.1 Challenges

The studies reviewed reveal that the advancement of driver behavior analysis has been achieved by incorporating machine learning, deep learning, sensor fusion, edge computing, and various other cutting-edge technologies. Several challenges, however, remain concerning the real-time deployment, scalability, reliability, and privacy of these systems. The major challenges identified from the literature are discussed below.

As shown in Figure 5, the major challenges of driver behavior analysis are data heterogeneity, driving conditions variability, model generalization, privacy and security, real-time processing, and lack of diverse and representative datasets. Due to the diversity of different driver behaviors, vehicles, road traffic settings, and driving situations, the reliable development of models and generalization is difficult. Furthermore, the vast amount of sensor and behavioral data creates a need for efficient data processing, privacy protection, and comprehensive assessment. These problems have been mentioned in recent studies, and the need for reliable validation, various data sets, comprehensible models, and scalable solutions for analyses in real-world scenarios has been pointed out (Mobini Seraji et al., 2025; Chai et al., 2024; Zhao et al., 2025).

**Figure 5:**
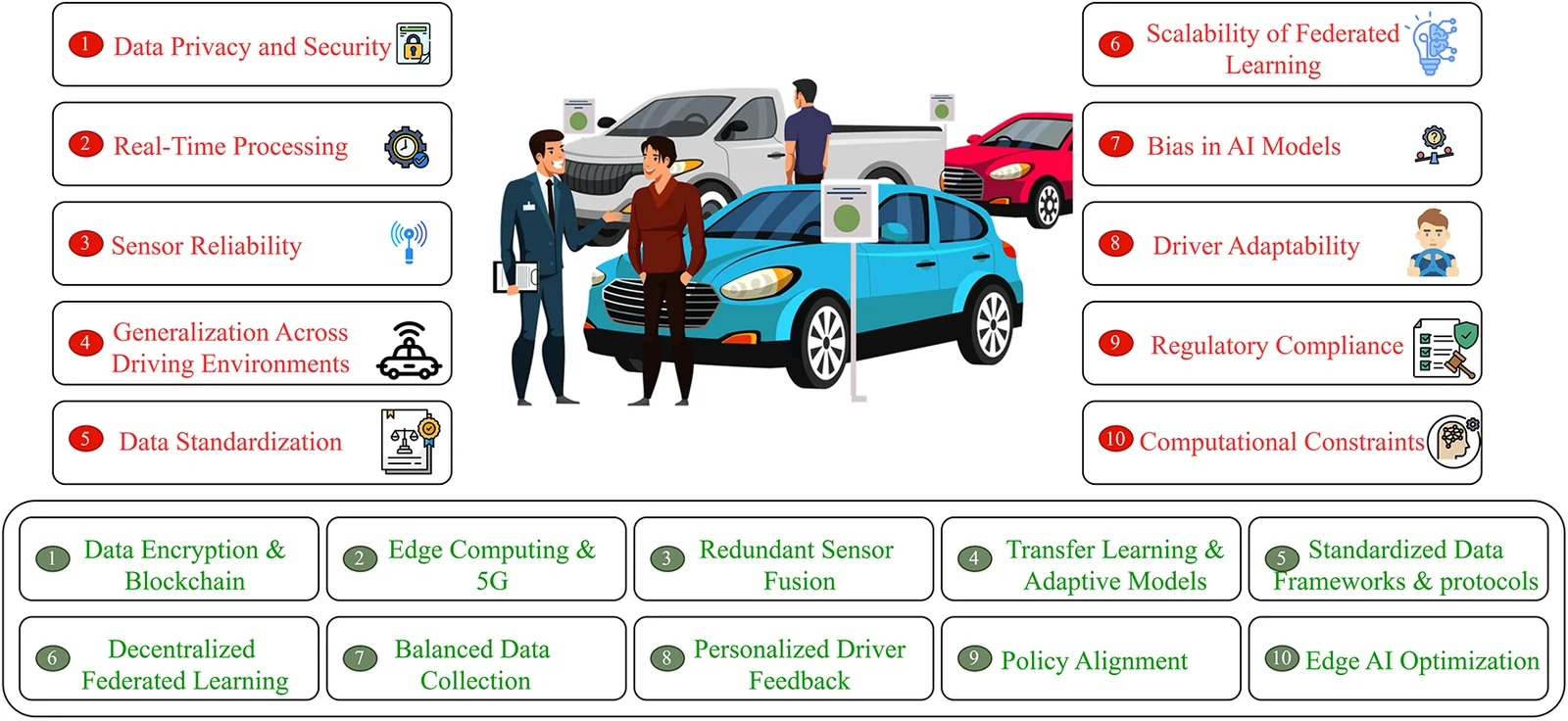
Challenges and solutions in vehicular driving data.

#### 7.1.1 Data privacy and security

The gathering and sending of driver behavior information creates grave issues of privacy and security (Costantino et al., 2022). Driving information may be sensitive and potentially subject to cyberattacks, unauthorized access, or data breaches, including real-time location, driving trends, and biometric data. In case such information gets into the wrong hands, it may be used to commit identity theft, commit insurance fraud, or conduct unauthorized surveillance.

Potential alternative solution: to increase the security level, access control can be introduced based on blockchain, which guarantees the impossibility of data manipulation and safe transmission. Furthermore, data transmission between vehicle sensors, cloud storage, and insurance companies can be encrypted and protected against interception.

#### 7.1.2 Real-time processing constraints

Real-time analysis of massive sensor data is computationally challenging, causing latency and performance problems (Jihong et al., 2024). Drivers need quick decision-making features for driver assistance, accident avoidance, and adaptive cruise control. Conventional cloud-based processing will cause delays, and real-time responses will be difficult.

Potential alternative solution: edge computing can be installed in vehicles, where data can be processed onboard using high-capacity computing devices, and only important information is sent to the cloud. Response time is also enhanced through the use of 5G networks, as the networks guarantee ultra-low latency in transmitting data.

#### 7.1.3 Sensor reliability and accuracy

The environmental conditions may influence the use of vehicle sensors, like LiDAR, cameras, and GPS (Gao et al., 2024). False alerts or erroneous driver risk evaluation may occur due to poor sensor readings, which will decrease the usefulness of AI models.

Potential alternative solution: multiple sensor data sources are combined together in sensor fusion techniques, which enhance accuracy through cross-checking of the readings. Noise and inconsistency can also be removed using AI-driven error correction models.

#### 7.1.4 Generalization across driving environments

Trained AI models on particular datasets might not be able to generalize to various road conditions, traffic types, and behaviors of drivers (Liu M. et al., 2024). When using the example of an AI model, it can be said that it will not work well in a rural or mountainous area, which was trained in the city.

Potential alternative solution: transfer learning enables AI models to generalize the knowledge gained from one dataset to another dataset. Also, self-learning adaptive models constantly revise themselves using the new driving data to improve the accuracy.

#### 7.1.5 Data standardization challenges

The data formats used by different car manufacturers and technology providers are different, thus making it hard to standardize the data on driver behavior across the platforms. This lack of standardization makes cooperation among insurance firms, authorities, and car producers difficult.

Potential alternative solution: industry-wide standardized data structures like the Automotive Grade Linux (AGL) and the Open Telematics Framework (OTF) offer the possibility of easily integrating the information provided by a number of various sources.

#### 7.1.6 Scalability in federated learning

Federated Learning (FL) allows one to train AI models on multiple heterogeneous devices without raw data exchange (Lu et al., 2024). Scalability is, however, a problem because network capabilities and computational power differ across vehicles.

Potential alternative solution: it is possible to optimize the decentralized FL architectures with asynchronous updates to make sure that models can be trained with the diverse fleets without saturating the single devices.

#### 7.1.7 Bias in AI models

AI models may be trained with bias, which results in misplaced or unfair risk estimates (Kuznietsov et al., 2024). As an illustration, the model, which is trained on certain demographics or driving behaviors, can subjugate some groups of drivers.

Potential Alternative Solution: Bias detection and fairness-conscious AI training is a potential resolution to issues because it guarantees that datasets are representative of various types of drivers and driving scenarios. Biases are also mitigated with the assistance of regular AI decision-making process audits.

#### 7.1.8 User acceptance and behavioral adaptation

Drivers will be reluctant to have AI-based behavior monitoring because of privacy issues, mistrust toward AI tests, or lack of incentives (Zeng et al., 2024). An intrusion that is perceived to be too invasive may result in bad user experiences and less adoption.

Resolution: the use of customized driver feedback systems that have gamification features can be implemented to motivate drivers to embrace safe driving behavior without loss of trust. Open AI decision-making is also better at making users accept.

#### 7.1.9 Regulatory compliance

The driver behavior analysis with the help of AI should be done in accordance with the different national and international laws regarding data privacy, liability, and socially responsible AI usage. These regulations are challenging to navigate, especially in cross-border vehicle operations (Koesdwiady et al., 2016).

Potential alternative solution: collaboration with policymakers through regulation means that AI models comply with legal and ethical guidelines. Institutions like the ISO 21448 safety standard in autonomous driving provide guidelines to comply with.

#### 7.1.10 Computational and energy constraints

Vehicles lack processing power and battery capacity, and it is not an easy task to conduct AI analysis in real-time. The execution of sophisticated AI models on vehicle embedded systems is likely to create performance bottlenecks and consume more energy (Liu et al., 2019).

Potential alternative solution: to address these issues, lightweight neural networks, model pruning, and hardware acceleration systems like TensorRT and edge AI chips can be used to make the AI models more performance-and energy-efficient. Furthermore, they can be deployed on specialized AI processors and other hardware with lower energy consumption, thereby minimizing the total energy usage without loss of real-time performance of AI models.

Real-time example: lightweight neural networks with hardware acceleration models such as TensorRT are optimized AI models that enhance efficiency and consume less energy.

Application: the DRIVE platform by NVIDIA streamlines AI-related computations in self-driving vehicles and makes use of the power-saving AI processing units to conduct real-time calculations without overloading the vehicle power supply.

Future work should be directed toward establishing an integrated driver behavior analysis framework based on identified behavioral tasks, which are based on a common taxonomy and analytical model. This framework can include multimodal data collection, edge-based processing, federated learning, secure aggregation, personalization, and real-time feedback. Future validation should use varying driver(s), road types, vehicle(s), and real-world data to assess accuracy, generalization, latency, privacy, and scalability. This integrated direction can help to build adaptive and privacy-preserving driver behavior analysis systems for real-time applications.

### 7.2 Proposed integrated architecture for driver behavior analysis

To address the aforementioned problems in integration, privacy, real-time processing, scalability, and personalization, the integrated architecture of driver behavior analysis is proposed, which is depicted in Figure 6. The integrated architecture integrates data collection, edge processing, federated learning, secure aggregation, personalization, and real-time feedback in a continuous process.

**Figure 6:**
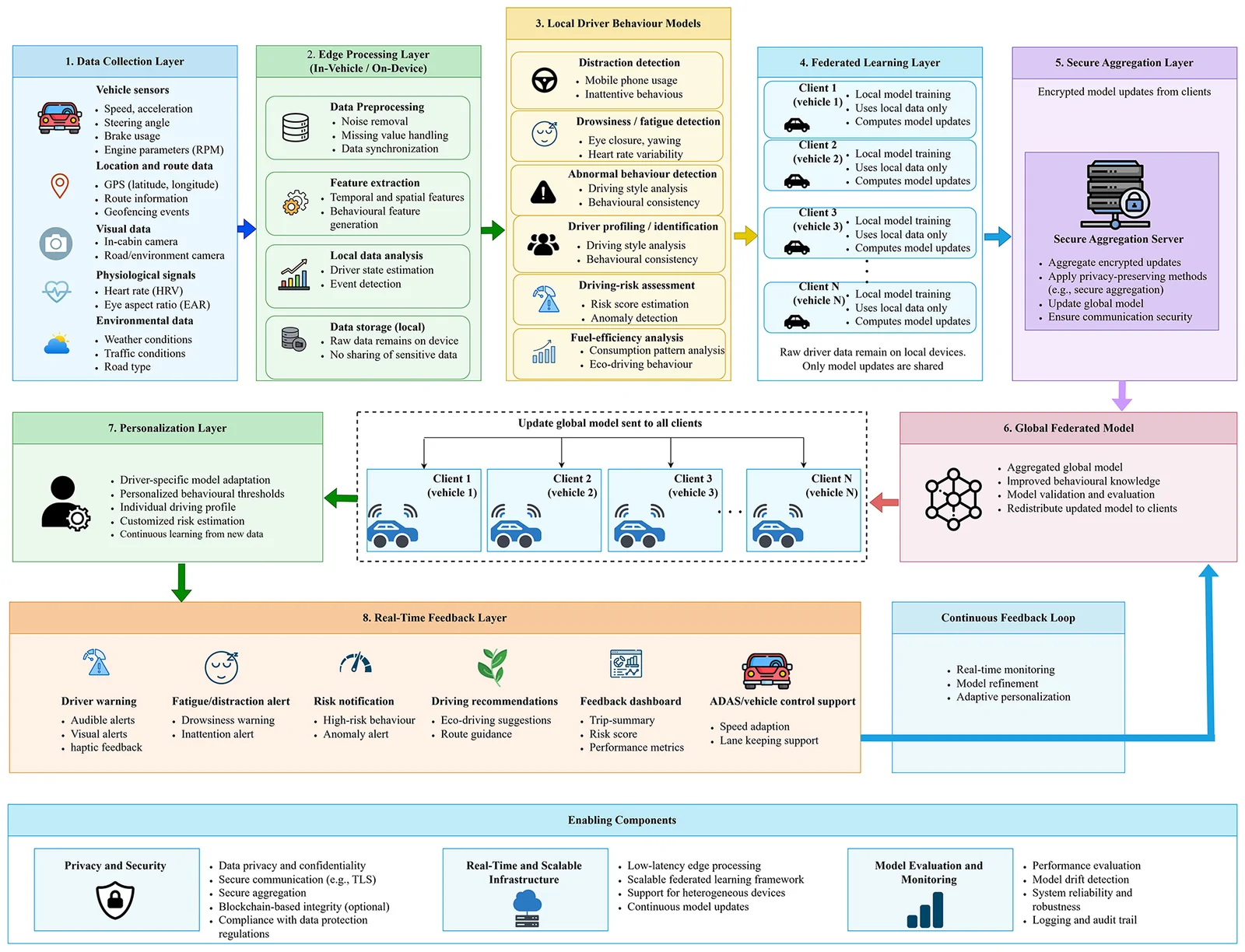
Proposed integrated architecture for driver behavior analysis.

The integrated architecture proposed in Figure 6 is composed of various layers to enable privacy-preserving and real-time driver behavior analysis. The Data Collection Layer collects information from a variety of sources such as vehicle sensors, GPS and route information, visual information, physiological information, and environmental information, including speed, acceleration, steering angle, brake usage, location information, heart rate, and traffic information. Local preprocessing operations like noise removal, missing-value handling, data synchronization, and feature extraction are performed by the Edge Processing Layer, which produces relevant temporal, spatial, and behavioral features while keeping sensitive raw data locally. The Local Driver Behavior Models Layer processes the data and analyses multiple behaviors such as driver distraction detection, drowsiness and fatigue detection, abnormal behavior detection, driver profiling and identification, driving-risk assessment, or fuel-efficiency data analysis. Federated learning layer: multiple vehicles/edge devices serve as federated clients and learn local models from the local data without transferring driver data; only the model updates are communicated. Participating clients send their model updates to the Secure Aggregation Layer, which aggregates the updates in a privacy-preserving manner to create an updated global model without revealing any sensitive information during communication. This Global Federated Model is subsequently transferred back to the respective participating clients, thus transferring the behavioral knowledge acquired in the various vehicles and driving situations to the following local training. The Personalization Layer is used to tailor the global model to individual driver characteristics, behavioral patterns, and risk profiles, to give each driver a more personalized analysis and recommendation. Finally, the Real-Time Feedback Layer translates the behavior-analyzed data into real-time, actionable outputs like driver warnings, fatigue or driver distraction alerts, risk notifications, eco-driving recommendations, feedback dashboards, and ADAS or vehicle-control support. This continuous feedback loop allows for the inclusion of the updated models and personalized information into the later behavior analysis, thus enabling continuous, scalable, privacy-respectful, and low-latency driver behavior monitoring.

## 8 Conclusion and future directions

This study analyzed recent research on vehicular driving pattern analysis and discussed major driver behavior tasks such as driving pattern recognition, driver distraction detection, abnormal behavior detection, driver profiling and identification, fuel-efficiency analysis, and driving-risk assessment. Machine learning, deep learning, sensor fusion, edge computing, and federated learning are identified as potential technologies for accurate and timely driver behavior analysis in the reviewed studies. However, current methods are mostly geared toward single behavioral tasks, and thus do not have a high level of integration between different analysis tasks. Data privacy, real-time processing, scalability, heterogeneous data, model generalization, and bias are also challenges. The proposed integrated architecture outlines the direction toward the possibilities of integrating data collection, edge processing, federated training, secure aggregation, personalization, and real-time feedback into one unified process. Future research should involve validation of such methods with a variety of drivers, vehicles, road types, and real-world data with respect to accuracy, latency, privacy, scalability, and generalization.

## Data Availability

The original contributions presented in the study are included in the article/Supplementary material, further inquiries can be directed to the corresponding author.
